# Deciphering the Epidemiological Characteristics and Molecular Features of *bla*_KPC–2_- or *bla*_NDM–1_-Positive *Klebsiella pneumoniae* Isolates in a Newly Established Hospital

**DOI:** 10.3389/fmicb.2021.741093

**Published:** 2021-11-10

**Authors:** Ruifei Chen, Ziyi Liu, Poshi Xu, Xinkun Qi, Shangshang Qin, Zhiqiang Wang, Ruichao Li

**Affiliations:** ^1^Department of Clinical Laboratory, Henan Provincial People’s Hospital, Department of Clinical Laboratory of Central China Fuwai Hospital, Central China Fuwai Hospital of Zhengzhou University, Zhengzhou, China; ^2^Jiangsu Co-innovation Center for Prevention and Control of Important Animal Infectious Diseases and Zoonoses, College of Veterinary Medicine, Yangzhou University, Yangzhou, China; ^3^Institute of Comparative Medicine, Yangzhou University, Yangzhou, China; ^4^School of Pharmaceutical Sciences, Zhengzhou University, Zhengzhou, China

**Keywords:** *bla*_KPC–2_, *bla*_NDM–1_, whole-genome sequencing, hypervirulence, novel structure, new hospital

## Abstract

The emergence of hypervirulent carbapenem-resistant *Klebsiella pneumoniae* (hv-CRKP) was regarded as an emerging threat in clinical settings. Here, we investigated the prevalence of CRKP strains among inpatients in a new hospital over 1 year since its inception with various techniques, and carried out a WGS-based phylogenetic study to dissect the genomic background of these isolates. The genomes of three representative *bla*_NDM–1_-positive strains and the plasmids of four *bla*_KPC–2_-positive strains were selected for Nanopore long-read sequencing to resolve the complicated MDR structures. Thirty-five CRKP strains were identified from 193 *K. pneumoniae* isolates, among which 30 strains (85.7%) harbored *bla*_KPC–2_, whereas the remaining five strains (14.3%) were positive for *bla*_NDM–1_. The antimicrobial resistance profiles of *bla*_NDM–1_-positive isolates were narrower than that of *bla*_KPC–2_-positive isolates. Five isolates including two *bla*_NDM–1_-positive isolates and three *bla*_KPC–2_-positive strains could successfully transfer the carbapenem resistance phenotype by conjugation. All CRKP strains were categorized into six known multilocus sequence types, with ST11 being the most prevalent type. Phylogenetic analysis demonstrated that the clonal spread of ST11 *bla*_KPC–2_-positive isolates and local polyclonal spread of *bla*_NDM–1_-positive isolates have existed in the hospital. The *bla*_NDM–1_ gene was located on IncX3, IncFIB/IncHI1B, and IncHI5-like plasmids, of which IncFIB/IncHI1B plasmid has a novel structure. By contrast, all ST11 isolates shared the similar *bla*_KPC–2_-bearing plasmid backbone, and 11 of them possessed pLVPK-like plasmids. In addition, *in silico* virulome analysis, *Galleria mellonella* larvae infection assay, and siderophore secretion revealed the hypervirulence potential of most *bla*_KPC–2_-positive strains. Given that these isolates also had remarkable environmental adaptability, targeted measures should be implemented to prevent the grave consequences caused by hv-CRKP strains in nosocomial settings.

## Introduction

*Klebsiella pneumoniae* is an important clinical pathogen that can cause severe hospital-acquired infections among immunocompromised patients (Pau et al., 2015). Nowadays, *K. pneumoniae* has evolved into two distinct pathotypes: hypervirulent *K. pneumoniae* and classical *K. pneumoniae* (cKp) (Shon et al., 2013). Both pathotypes are global challenges for nosocomial infections (Patel et al., 2014). Classical *K. pneumoniae* is capable of acquiring various antimicrobial resistance (AMR) genes, resulting in the emergence of multidrug-resistant (MDR) and extensively drug-resistant (XDR) strains (Navon-Venezia et al., 2017). The typical representation is carbapenem-resistant *K. pneumoniae* (CRKP). Hypervirulent *K. pneumoniae* can cause infections, such as liver abscesses, pneumoniae, meningitis, and endophthalmitis in healthy individuals, and the *rmpA* and *rmpA2* genes are associated with its pathogenicity (Shon et al., 2013). For a long time, *K. pneumoniae* did not simultaneously encode the phenotypes of MDR and hypervirulence (Yang et al., 2020b). However, in recent years, the convergence of carbapenem resistance and virulence in a single epidemic clone has been reported constantly, which becomes a serious public health issue (Chen and Kreiswirth, 2018; Wong et al., 2018a; Xie et al., 2020). The most representative clade is ST11-CR-HvKp detected in different regions of China (Wong et al., 2018b; Yao et al., 2018; Xu et al., 2019). A recent study successfully traced ST11-CR-HvKp and speculated that the stool may be a reservoir of it (Zheng et al., 2020). These findings revealed the subsistent dissemination of this clone among nosocomial systems.

Currently, *K. pneumoniae* carbapenemase (KPC) is one of the most clinically significant carbapenemase, and its rapid dissemination has become a public health threat globally (Chen et al., 2014). To date, 95 KPC variants have been identified^1^. The pandemic of KPC-producing *K. pneumoniae* is dominated by clonal group 258, which consists of ST258 and its single-locus variants ST11, ST340, and ST512 (Chen et al., 2014). ST258 is the major KPC-producing *K. pneumoniae* sequence type (ST) in North America, Latin America, and several countries in Europe, whereas ST11 prevails mainly in Asia and Latin America (Munoz-Price et al., 2013; Andrade et al., 2014). In China, a study revealed that *bla*_KPC–2_ was presented in 71% of 109 ertapenem-resistant *K. pneumoniae* isolates in a teaching hospital in Shanghai, and it was often detected along with CTX-M type ESBL enzymes (Chen et al., 2011). Besides, a retrospective observational study (2008–2018) of clinical CRKP isolates found the main CRKP ST was ST11, and *bla*_KPC–2_ was the most prevalent variant in Zhejiang, China (Hu et al., 2020). In addition, the *bla*_NDM_-positive *K. pneumoniae* is another target for nosocomial infection control, which colonized in hospitals of China with high incidence (Qin et al., 2014). Therefore, it is necessary to recognize the dissemination characteristics and molecular features of CRKP. As the most prevalent area of CRKP, the detection rate of CRKP in Henan province reached 32.8% in 2019 according to China Antimicrobial Surveillance Network 2019 annual report^2^. Nevertheless, the epidemiological investigation of CRKP in newly established hospitals is still limited. To systematically study, the prevalence and transmission of CRKP in new hospitals are of guiding significance to evaluate the development trend of CRKP; hence, we aim to investigate the prevalence and genomic characterization of CRKP in a newly established hospital in China and further explore the underlying risk factors, viability, virulence, antibiotic resistance profiles and molecular characteristics of CRKP.

## Materials and Methods

### Research Design

During March 2018–August 2019, seven kinds of samples including blood, ascitic fluid, sputum, bronchoalveolar fluid, wound secretion, urine, and ductus venosus of inpatients were collected from either public wards or intensive care units (ICUs) in a newly established hospital in Henan, China. The hospital that specializes in the treatment of cardiovascular diseases is a 1,000-bed tertiary hospital with 132 ICU beds and 34 public wards. Besides, the present study was approved by the Research Ethics Committee of Henan Provincial People’s Hospital.

### Bacterial Isolation and Identification

Collected samples were subjected to standard bacterial isolation procedure. The samples were streaked directly onto 5% sheep blood agar plate. Colonies of different morphologies were selected to perform subsequent purification and stocked at −80°C. The detection of carbapenemase-encoding genes was conducted by multiplex polymerase chain reaction (PCR) (Poirel et al., 2011; Supplementary Table 1), and laboratory-stored strains carrying the corresponding carbapenemase-encoding genes were used as the positive control. Species identification of the strains and subsequent antimicrobial susceptibility testing were conducted by BD Phoenix100 (Becton, Dickinson and Company, Franklin Lakes, NJ, United States) and verified by disk diffusion method. The minimum inhibitory concentrations of ciprofloxacin, levofloxacin, aztreonam, chloramphenicol, ampicillin, ampicillin–sulbactam, piperacillin, piperacillin–tazobactam, amoxicillin–clavulanic acid, gentamicin, amikacin, cefazolin, ceftazidime, cefotaxime, cefepime, meropenem, imipenem, trimethoprim-sulfamethoxazole, and tetracycline were interpreted based on the standard of the Clinical and Laboratory Standards Institute [CLSI] (2018) except tigecycline and polymyxin B, which followed the criteria of European Committee on Antimicrobial Susceptibility Testing (version 11.0)^3^. *Escherichia coli* ATCC25922 was used as the quality control strain.

### Characterization of STs, Capsular Types, Virulence Genes, and Virulence Phenotype

To preliminarily distinguish the STs and capsular types and confirm the presence of the virulence-associated genes including *rmpA*, *rmpA2*, *iroN*, and *iutA*, multiplex PCR analysis was performed as previously mentioned (Yu et al., 2018; Supplementary Table 1), and laboratory-stored strains carrying the corresponding genes were used as the positive control. Furthermore, the hypervirulence phenotype of *K. pneumoniae* was evaluated using string test and *Galleria mellonella* larvae infection assay. For string test, all isolates were inoculated onto 5% sheep blood agar and incubated at 37°C, and the cutoff criterion for positive was the viscous string longer than 5 mm (Shon et al., 2013). For *G. mellonella* larvae infection assay, larvae of approximately 300 mg were stored in a special box at 4°C until being used. Overnight cultures of *K. pneumoniae* were washed and adjusted to 10^6^ colony-forming units (CFU)/mL using phosphate-buffered saline (PBS). Ten larvae in each group were challenged with 10 μL of diluents, with ST11 clinical cKP HS11286 derivate YZ6 (Xie et al., 2018) used as the negative control. Infected larvae were incubated in sterilized Petri dishes at 37°C for 72 h, and survival rate was recorded every 24 h. All experiments were repeated in triplicate.

### Filter Mating Assay

Transferability of carbapenem resistance phenotype was determined using conjugation assay with a filter mating method. Thirty-five CRKPs were used as donor strains, and *K. pneumoniae* YZ6 Hyg^r^ was served as the recipient strain. Transconjugants were selected on LB agar plates supplemented with hygromycin (200 mg/L) and meropenem (2 mg/L). The transconjugants harboring carbapenemase encoding genes were confirmed by PCR and antimicrobial susceptibility testing.

### Growth Curves

To investigate the fitness of CRKP isolates, growth curves of seventeen strains including 15 CRKP isolates in this study, YZ6 and ATCC700603 in LB broth were conducted according to standardized protocols using three technical replicates and three biological replicates. *Klebsiella pneumoniae* ATCC700603 and YZ6 were regarded as control strains (Schaufler et al., 2016). Growth rates were calculated as follows: μ = (ln(CFU/mL t1) – ln(CFU/mL t0))/t1 – t0.

### Siderophore Secretion

We qualitatively detected siderophore secretion of CRKP isolates as previously described (Schwyn and Neilands, 1987). A single colony was transplanted into MKB solid medium for iron starvation treatment. After incubation for 24 h at 37°C, the bacterial suspension was adjusted to an OD600 of 0.6 by normal saline, and then 5 μL of suspension was placed on agar plates containing chrome azurol S-iron(III)-hexadecyltrimethylammonium bromide and incubated overnight at 37°C. The orange secretory ring around the colony indicated the production of siderophore. *Klebsiella pneumoniae* ATCC700603 and YZ6 were considered as control strains, and the experiment was repeated three times for each strain.

### Biofilm Formation

Biofilm formation assays were conducted as previously mentioned (Ma et al., 2020). Overnight cultures of tested isolates were adjusted to a cell density equivalent to a 0.5 McFarland standard. Two hundred microliters of culture per well were transferred to a 96-well plate. After incubation at 37°C for 2 days, cultures were discarded, and wells were washed twice with 200 μL PBS. The biofilms were fixed in methanol for 10 min. Subsequently, wells were stained with 1% crystal violet solution for 10 min and rinsed with PBS until colorless. Finally, biofilms were dissolved in 100 μL of 30% formic acid for 30 min, and biofilm formation was quantified by measuring the absorbance at OD590. *Klebsiella pneumoniae* ATCC700603 and YZ6 were used as control strains.

### Human Serum Resistance

We evaluated the ability of human serum resistance as previously described (Heiden et al., 2020). Briefly, 5 μL of overnight culture was added to 495 μL LB fresh medium and incubated for 1.5 h at 37°C. Inoculum was resuspended with 1 mL of sterile 1 × PBS. Thirty microliters was mixed in triplicates with 270 μL 50% human serum in 96-well plates. Meanwhile, 30-μL mixture was sucked out from each well, serially diluted, placed on LB agar, and counted the next day. After incubation for 4 h at 37°C, 30 μL of mixture was subjected to the same procedure. Finally, the number of colonies of 0- and 4-h time points was compared to evaluate the survival ability of CRKP isolates in human serum. *Klebsiella pneumoniae* ATCC700603 and YZ6 were used as control strains.

### Desiccation Resilience

Desiccation resilience assays were carried out according to previously methods (Heiden et al., 2020) with minor modified. Briefly, a single colony was cultured in LB broth until bacterial cells reached an OD600 value of 0.6–0.8. One hundred microliters of inoculum was serially diluted, plated on LB agar plates, and counted the next day. Meanwhile, another 100-μL inoculum was transferred to 96-well plates. Then, the plates were laid flat in a glass sterile dryer supplemented with desiccant and placed in a 37°C incubator. After 6 days of drying, 100 μL/well fresh LB broth was readded in 96-well plates; the prepared 96-well plates were cultured with 200-rpm shaking at 37°C for 3 h. At this point, 100 μL was collected, and the same procedure was performed to count the number of colonies. *Klebsiella pneumoniae* ATCC700603 and YZ6 were used as control strains.

### Statistical Analysis

The data were presented using GraphPad Prism 8.3.0. After ensuring that the data were non–normally distributed, the non-parametric Kruskal–Wallis test was utilized to perform multiple comparisons among different groups. Bonferroni adjustment was applied; the corrected *p* < 0.1 was considered significant.

### Genome Extraction and High-Throughput Sequencing

Genomic DNA of the 35 CRKP strains was extracted using the TIANamp bacterial DNA kit (TianGen, Beijing, China). The plasmids of four ST11 *bla*_KPC–2_-positive strains, which were selected based on virulence test (C13, C26, C31, and C38), were extracted using the Qiagen plasmid midi-kit (Qiagen, Germany). The extracted genomic DNA was evaluated by 1% agarose gel electrophoresis and quantified by the Qubit fluorometer and then subjected to short-read sequencing (2 × 150 bp) with the Illumina HiSeq 2500 platform. Subsequently, genomic DNAs of three *bla*_NDM_-positive strains (C11, C39, and C20) from different branches and plasmids of four aforementioned strains were sequenced with the Oxford Nanopore Technologies MinION long-read platform with the RBK004 barcoding library preparation kit and MinION R9.4.1 flow cells as previously described (Wick et al., 2017; Li et al., 2018).

### Bioinformatics Analysis and Phylogenomic Tree Construction

The short-read Illumina raw sequences of CRKP were quality filtered and assembled by SPAdes (Bankevich et al., 2012), and contigs less than 500 bp were discarded. The clone lineages, STs, insertion sequences, AMR determinants, and the virulence genes of CRKP were identified using online tools^4^ and Kleborate tool (Wick et al., 2018). The phylogenetic trees of the comparison within CRKP in this study and the comparison between CRKP in this study and other strains in GenBank were constructed using Roary and FastTree based on SNPs of core genomes (Price et al., 2009; Page et al., 2015), and further visualization and modification were performed in iTOL^5^. Combining the formed tree file and the gene presence and absence file, a phylogenetic tree with a matrix describing the presence and absence of core and accessory genes was constructed. The sequences of 35 CRKP were compared against the classical virulence plasmid pLVPK (GenBank accession AY378100), and representative plasmid sequences were further plotted by GView web server^6^ using pLVPK as reference sequence. The layout and output were edited in the GView Java stand-alone application obtained from results webpage. Genomic DNA with short-read Illumina and long-read Nanopore data was subjected to perform *de novo* hybrid assembly as described previously (Wick et al., 2017). The complete genome sequences were annotated using RAST^7^ automatically and modified manually. BRIG and Easyfig were used to generate the genetic comparison figures (Alikhan et al., 2011).

### Risk Factor Analysis

To analyze the risk factors responsible for the occurrence of CRKP, the clinical information of CRKP-carriers was compared to the non-carriers in terms of underwent different variables, which included gender, age, ICU, exposure to carbapenem during hospital stay, isolation season, and sample type. For all data, logistic regression analysis models were used to obtain odds ratios (ORs) and 95% confidence intervals (CIs) for analysis of independent risk factors associated with the occurrence of CRKP. Categorical variables were compared using χ^2^ test or two-tailed Fisher exact test, with *p* < 0.05 considered statistically significant. All statistical analyses were processed in SPSS version 22.0.

### Data Availability

The draft genome sequences of 32 CRKP isolates have been deposited in the GenBank database under BioProject accession no. PRJNA705380. The complete genome sequences of three *bla*_NDM–1_-positive CRKP isolates obtained by hybrid assembly have been deposited in GenBank with accession numbers C11 (pending, deposited in figshare database temporarily), C20 (CP084103-CP084106), and C39 (CP061700-CP061702). The assembled plasmid sequences of four strains (C13, C26, C31, and C38) were deposited in the figshare database (https://doi.org/10.6084/m9.figshare.14199287.v5) for reference. Additional data that support the findings of this study are available from the corresponding authors upon reasonable request.

## Results

### Characterization of Carbapenem-Resistant *Klebsiella pneumoniae*, Resistance Phenotypes, and Transferability

**FIGURE 1:**
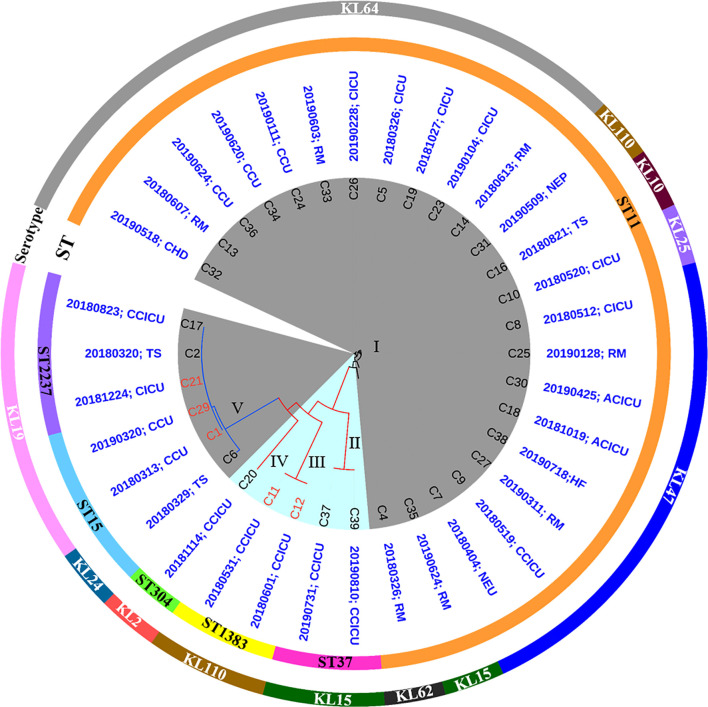
Phylogenetic analysis of 35 CRKP isolates and their basic characterization. *bla*_NDM–1_- and *bla*_KPC–2_-positive strains are highlighted in blue and gray background, respectively. Circles outside the tree indicate the STs and serotypes of each strain. The isolation time and sites are shown in blue words (RM, respiratory medicine ward; CHD, coronary heart disease ward; CCU, coronary care unit; CICU, comprehensive intensive care unit; NEP, nephrology ward; TS, thoracic surgery ward; ACICU, adult cardiac intensive care unit; HF, heart failure ward; NEU, neurology ward; CCICU, children cardiac intensive care unit). The labels marked in red represent the carbapenem resistance phenotype could successfully transfer by conjugation.

From March 2018 to August 2019, a total of 1,413 isolates were collected from different wards or ICUs of a newly established hospital in Henan province, China. In these isolates, *K. pneumoniae* (193, 14%) was the most prevalent species, followed by *Acinetobacter baumannii* [177 (13%)], *Pseudomonas aeruginosa* [158 (11%)], *E. coli* [116 (8%)], and *Staphylococcus aureus* [92 (7%)], which were the common nosocomial pathogens (Supplementary Table 2). *Klebsiella pneumoniae* isolates were from 18 different wards or ICUs in the hospital (Supplementary Table 3). PCR and Sanger sequencing identified 35 [of 193 (18.1%)] carbapenemase-producing *K. pneumoniae*. Among them, 30 isolates were positive for *bla*_KPC–2_, whereas the remaining five isolates carried *bla*_NDM–1_. All strains exhibited resistance to tested β-lactam antibiotics meropenem, imipenem, aztreonam, ampicillin, ampicillin–sulbactam, piperacillin, piperacillin–tazobactam, amoxicillin–clavulanic acid, cefazolin, ceftazidime, cefotaxime, and cefotaxime. Meanwhile, most strains were resistant to ciprofloxacin [30/35 (85.7%)], levofloxacin [30/35 (85.7%)], chloramphenicol [14/35 (40%)], gentamicin [33/35 (94.3%)], amikacin [25/35 (71.4%)], trimethoprim-sulfamethoxazole [21/35 (60%)], and tetracycline [20/35 (57.1%)], but remained susceptible to tigecycline [34/35 (97.1%)] and polymyxin B [35/35 (100%)] (Supplementary Table 4). To investigate the transferability of the carbapenemase-encoding genes, 35 strains were subjected to conjugation assay. However, only five isolates (C11, C12, C1, C29, and C21) including two *bla*_NDM–1_-positive strains and three *bla*_KPC–2_-positive strains could successfully transfer the carbapenem resistance phenotype to the recipient strain YZ6 Hyg^R^, suggesting the carbapenemase-encoding genes of them were located on conjugative plasmids (Figure 1).

### Risk Factors Associated With the Emergence of Carbapenem-Resistant *Klebsiella pneumoniae*

To evaluate the risk factors accounting for the emergence of CRKP, clinical information without individual identification of patients was compared (Supplementary Tables 5, 6). Among 35 CRKP colonization cases ranged from 3 days to 80 years old, 66% (23/35) were male and 71.4% (25/35) were older than 50 years, suggesting CRKP tended to invade middle-aged and elder patients. Furthermore, colonization was observed in 10 different wards or ICUs. Among them, 60% (21/35) of cases were from ICUs. Besides, 71.4% (25/35) of patients used to experience carbapenem treatment. Comparing with the CRKP non-carriers, no significant differences were detected in gender, age, isolation season, ICU patients, and sample source groups, but the correlation was detected between carbapenem treatment history and being CRKP positive (*p* = 0.009) [OR = 3.453 (95% CI = 1.369–8.707)].

### Multilocus ST Genotyping, Serotypes, and Phylogenetic Analysis of Carbapenem-Resistant *Klebsiella pneumoniae* Isolates

Analysis of genomic characteristics revealed that the *bla*_KPC–2_-positive isolates belonged to three known STs (ST11, ST15, and ST2237), with ST11 being the most dominant type [24/30 (80%)]. In contrast to the *bla*_KPC–2_-positive isolates, five *bla*_NDM–1_-positive strains exhibited diverse STs including ST37, ST1383, and ST304. To our knowledge, ST1383 and ST304 *K. pneumoniae* strains were not associated with carbapenem resistance; especially, the ST304 type has never been reported to carry *bla*_NDM–1_. Analysis of *wzi* locus revealed that seven different types (KL64, KL110, KL10, KL25, KL47, KL15, and KL62) existed in ST11 isolates, whereas KL24 and KL19 were identified in ST15 and ST2237 isolates. In addition, *bla*_NDM–1_-positive strains possessed three serotypes including KL15, KL110, and KL2. Roary identified a total of 9,610 genes in pangenome, including core genes (*n* = 3,951), soft core genes (*n* = 156), shell genes (*n* = 2,100), and cloud genes (*n* = 3,403) (Supplementary Figures 1, 2). A maximum likelihood phylogenetic tree demonstrated that all strains were clustered into five clades. ST11 *bla*_KPC–2_-positive isolates were grouped into cluster I, whereas the remaining *bla*_KPC–2_-positive isolates including three ST15 strains and three ST2237 strains were assigned to cluster V, suggesting the clonal expansion of *bla*_KPC–2_-positive isolates dominated by ST11 *K. pneumoniae* along with ST15 and ST2237 *K. pneumoniae* that existed in this hospital. By contrast, five *bla*_NDM_ positive isolates were classified into cluster II (two ST37 strains), III (two ST1383 strains), and IV (one ST304 strain), respectively. The diversity of multilocus ST showed that *bla*_NDM_-carrying strains had polyclonal spread. However, the epidemic features were distinguished between *bla*_KPC–2_-harboring and *bla*_NDM–1_-harboring isolates, as the *bla*_KPC–2_-harboring isolates were detected in 10 different wards and ICUs, whereas the *bla*_NDM–1_ strains were solely concentrated in children cardiac ICU. These findings suggested that the clonal spread of ST11 *bla*_KPC–2_-positive isolates and local polyclonal spread of *bla*_NDM–1_-positive isolates have existed in this hospital (Figure 1). Furthermore, the ST11 and ST15 CRKP strains reported in other studies (Liu et al., 2012; Li et al., 2020; Zheng et al., 2020) were also highly related to corresponding strains in this study. These strains derived from different hospitals in China, suggesting the CRKP involved in this study has been widely spread among the clinical settings (Supplementary Figure 3).

### Resistome Analysis of Carbapenem-Resistant *Klebsiella pneumoniae* Isolates

Resistome analysis revealed that the *bla*_KPC–2_-positive strains harbored more types of AMR genes than those found in *bla*_NDM–1_-positive strains. Moreover, *bla*_KPC–2_-positive strains possessed almost all classes of genes conferring resistance to aminoglycoside, quinolone, sulfonamide, fosfomycin, tetracycline, and β-lactam, with the most prevalent being *fosA, bla*_TEM–1B_, *rmtB*, *aadA2*, and *bla*_CTX–M–65_ genes, which implied that *bla*_KPC–2_ had potential risks of cotransmission with other AMR genes. However, the AMR profiles of *bla*_NDM–1_-positive isolates were narrower than those of *bla*_KPC–2_-positive isolates. They were resistant to β-lactam but still susceptible to other antibiotics including ciprofloxacin, levofloxacin, amikacin, polymyxin B, and tigecycline. Interestingly, three *bla*_NDM–1_-positive strains carried rare ESBLs *bla*_SFO–1_-like (with three bases mutation compared to *bla*_SFO–1_) and *bla*_VEB–3_, which were usually excluded from routine surveillance (Table 1).

**TABLE 1:** Summary of all carbapenem-resistant *Klebsiella pneumoniae* strains revealed by WGS data and virulence assay in this study.

Strains	STs/capsular types	Antimicrobial resistance genes	Virulence genes	String test	*Galleria mellonella* larvae infection (survival rate at 72 h)	Virulence score^b^
C13	ST11/KL64	*qnrS1*, *aadA2*, *rmtB*, *bla*_CTX–M–65_, *bla*_KPC–2_, *bla*_SHV–12_, *bla*_TEM–1B_, *catA2*, *dfrA14*, *fosA*, *sul2*, *tet*(A)	*Yersiniabactin*, *aerobactin*, *rmpA*, *rmpA2*	Negative	20%	4
C32	ST11/KL64	*qnrS1*, *rmtB*, *bla*_CTX–M–65_, *bla*_KPC–2_, *bla*_SHV–12_, *bla*_TEM–1B_, *catA2*, *fosA*, *sul2*, *tet*(A)	*Yersiniabactin*, *aerobactin*, *rmpA*, *rmpA2*	Negative	—^a^	4
C34	ST11/KL64	*qnrS1*, *aadA2*, *rmtB*, *bla*_CTX–M–65_, *bla*_KPC–2_, *bla*_SHV–12_, *bla*_TEM–1B_, *dfrA14*, *fosA*, *sul2*, *tet*(A)	*Yersiniabactin*, *aerobactin*, *rmpA*, *rmpA2*	Negative	—	4
C36	ST11/KL64	*qnrS1*, *aadA2*, *rmtB*, *bla*_CTX–M–65_, *bla*_KPC–2_, *bla*_SHV–12_, *bla*_TEM–1B_, *dfrA14*, *fosA*, *sul2*, *tet*(A)	*Yersiniabactin*, *aerobactin*, *rmpA*, *rmpA2*	Negative	—	4
C24	ST11/KL64	*qnrS1*, *aadA2*, *rmtB*, *bla*_CTX–M–65_, *bla*_KPC–2_, *bla*_SHV–12_, *bla*_TEM–1B_, *catA2*, *dfrA14*, *fosA*, *sul2*, *tet*(A)	*Yersiniabactin*, *aerobactin*, *rmpA*, *rmpA2*	Negative	—	4
C33	ST11/KL64	*qnrS1*, *aadA2*, *bla*_CTX–M–65_, *bla*_KPC–2_, *bla*_SHV–11_, *fosA*, *sul2*, *tet*(A)	*Yersiniabactin*, *aerobactin*, *rmpA*, *rmpA2*	Negative	—	4
C26	ST11/KL64	*qnrS1*, *rmtB*, *bla*_CTX–M–65_, *bla*_KPC–2_, *bla*_SHV–12_, *bla*_TEM–1B_, *catA2*, *dfrA14*, *fosA*, *tet*(A)	*Yersiniabactin*, *aerobactin*, *rmpA2*	Positive	0%	4
C5	ST11/KL64	*qnrB4*, *aac(3)-IId*, *aadA2*, *armA*, *bla*_DHA–1_, *bla*_KPC–2_, *bla*_SHV–11_, *bla*_TEM–1B_, *catA2*, *fosA*, *mph*(A), *mph*(E), *msr*(E),	*Yersiniabactin*, *aerobactin*, *rmpA2*	Negative	—	4
C19	ST11/KL64	*aadA2*, *rmtB*, *bla*_CTX–M–65_, *bla*_KPC–2_, *bla*_SHV–12_, *bla*_TEM–1B_, *fosA*	*Yersiniabactin*, *aerobactin*, *Salmonchelin*, *rmpA*, *rmpA2*	Negative	0%	4
C14	ST11/KL64	*aadA2*, *rmtB*, *bla*_CTX–M–65_, *bla*_KPC–2_, *bla*_SHV–12_, *bla*_TEM–1B_, *fosA*	*Yersiniabactin*, *aerobactin*, *Salmonchelin*, *rmpA*, *rmpA2*	Negative	0%	4
C23	ST11/KL64	*aadA2*, *rmtB*, *bla*_CTX–M–65_, *bla*_KPC–2_, *bla*_SHV–12_, *bla*_TEM–1B_, *fosA, dfrA12, mph*(A)	*Yersiniabactin*, *aerobactin*, *rmpA2*	Negative	—	4
C31	ST11/KL110	*aadA2*, *rmtB*, *bla*_CTX–M–65_, *bla*_KPC–2_, *bla*_SHV–12_, *bla*_TEM–1B_, *fosA, dfrA12, mph*(A)	*Yersiniabactin*, *aerobactin*, *Salmonchelin*, *rmpA*, *rmpA2*	Negative	0%	4
C16	ST11/KL10	*ARR-3*, *oqxA*, *oqxB*, *aac(3)-IId*, *aac(6′)Ib*, *aadA16*, *aadA2*, *aph(3′)-Ia*, *strA*, *strB*, *bla*_CTX–M–15_, *bla*_KPC–2_, *bla*_SHV–11_, *dfrA27*, *fosA*, *mph*(A), *tet*(A)	*Yersiniabactin*	Negative	—	1
C10	ST11/KL25	*qnrS1*, *oqxA*, *oqxB*, *aac(3)-IId*, *aadA2*, *rmtB*, *strA*, *strB*, *bla*_CTX–M–65_, *bla*_KPC–2_, *bla*_SHV–11_, *bla*_TEM–1B_, *catA2*, *dfrA14*, *fosA*, *sul2*, *tet*(A), *tet*(D)	*Yersiniabactin*	Negative	—	1
C8	ST11/KL47	*oqxA*, *oqxB*, *aadA2*, *rmtB*, *bla*_CTX–M–65_, *bla*_KPC–2_, *bla*_SHV–11_, *bla*_TEM–1B_, *catA2*, *fosA*	*Yersiniabactin*	Negative	—	1
C25	ST11/KL47	*qnrB6*, *qnrS1*, *bla*_CTX–M–3_, *bla*_KPC–2_, *bla*_SHV–11_, *bla*_TEM–1B_, *dfrA14*, *fosA*	*Yersiniabactin*	Negative	—	1
C18	ST11/KL47	*oqxA*, *oqxB*, *rmtB*, *bla*_CTX–M–65_, *bla*_KPC–2_, *bla*_SHV–12_, *bla*_TEM–1B_, *catA2*, *fosA*	*Yersiniabactin*, *aerobactin*, *rmpA2*	Negative	—	4
C30	ST11/KL47	*oqxA*, *oqxB*, *aadA2*, *rmtB*, *bla*_CTX–M–65_, *bla*_KPC–2_, *bla*_SHV–11_, *bla*_TEM–1B_, *catA2*, *fosA*, *mph*(E)	*Yersiniabactin*, *aerobactin*, *rmpA2*	Negative	—	4
C27	ST11/KL47	*oqxA*, *oqxB*, *aac(6′)Ib, aadA2*, *aph(3′)-Ia*, *bla*_CTX–M–15_, *bla*_CTX–M–65_, *bla*_KPC–2_, *bla*_OXA–1_, *bla*_SHV–11_, *bla*_TEM–1B_, *catA2*, *fosA*, *tet*(A)	*Yersiniabactin*, *aerobactin*, *rmpA2*	Negative	—	4
C38	ST11/KL47	*oqxA*, *oqxB*, *aac(6′)Ib, rmtB*, *aph(3′)-Ia*, *bla*_CTX–M–15_, *bla*_CTX–M–65_, *bla*_KPC–2_, *bla*_OXA–1_, *bla*_SHV–11_, *bla*_TEM–1B_, *catA2*, *fosA*, *tet*(A)	*Yersiniabactin*, *aerobactin*, *rmpA2*	Negative	0%	4
C7	ST11/KL47	*oqxA*, *oqxB*, *aac(6′)Ib, aadA2*, *rmtB*, *aph(3′)-Ia*, *bla*_CTX–M–15_, *bla*_CTX–M–65_, *bla*_KPC–2_, *bla*_OXA–1_, *bla*_SHV–11_, *bla*_TEM–1B_, *fosA*, *tet*(A)	*Yersiniabactin*, *aerobactin*, *rmpA2*	Negative	—	4
C9	ST11/KL47	*oqxA*, *oqxB*, *aac(6′)Ib, rmtB*, *aph(3′)-Ia*, *bla*_CTX–M–65_, *bla*_KPC–2_, *bla*_OXA–1_, *bla*_SHV–11_, *bla*_TEM–1B_, *fosA*, *tet*(A)	*Yersiniabactin*, *aerobactin*, *rmpA2*	Negative	—	4
C35	ST11/KL15	*oqxA*, *oqxB*, *aac(3)-IId*, *bla*_KPC–2_, *bla*_SHV–11_, *fosA*, *tet*(D)	*Yersiniabactin*	Negative	—	1
C4	ST11/KL62	*ARR-3*, *oqxA*, *oqxB*, *aac(3)-IId*, *aadA16*, *strA*, *strB*, *bla*_CTX–M–14_, *bla*_KPC–2_, *bla*_SHV–11_, *bla*_TEM–1B_, *dfrA27*, *fosA*, *mph*(A), *sul1*, *sul2*, *tet*(D)	*Yersiniabactin*	Negative	—	1
C37	ST37/KL15	*ARR-3*, *qnrA7*, *oqxA*, *oqxB*, *aac(3)-IId*, *bla*_NDM–1_, *bla*_SFO–1_-like, *bla*_SHV–11_, *bla*_TEM–1B_, *bla*_VEB–3_, *dfrA27*, *fosA*, *mph*(A), *sul1*	*Yersiniabactin*	Negative	—	1
C39	ST37/KL15	*ARR-3*, *qnrA7*, *oqxA*, *oqxB*, *aac(3)-IId*, *bla*_NDM–1_, *bla*_SFO–1_-like, *bla*_SHV–11_, *bla*_TEM–1B_, *bla*_VEB–3_, *dfrA27*, *fosA*, *mph*(A), *sul1*	*Yersiniabactin*	Negative	—	1
C11	ST1383/KL110	*oqxA*, *oqxB bla*_NDM–1_, *bla*_SHV–11_, *fosA*, *bla*_SHV–12_	None	Negative	—	0
C12	ST1383/KL110	*oqxA*, *oqxB bla*_NDM–1_, *bla*_SHV–11_, *fosA*, *bla*_SHV–12_	None	Negative	—	0
C20	ST304/KL2	*qnrS1*, *oqxA*, *oqxB*, *aac(3)-IId*, *aadA2*, *bla*_CTX–M–14_, *bla*_NDM–1_, *bla*_SHV–11_, *fosA*	*Yersiniabactin*	Negative	—	1
C6	ST15/KL24	*qnrB4*, *oqxA*, *oqxB*, *aac(6′)-IIa*, aadA2, *armA*, *bla*_KPC–2_, *bla*_LEN15_, *dfrA14*, *fosA*, *mph*(E), *msr*(E), *sul1*, *sul2*	*Yersiniabactin*, *aerobactin*, *rmpA2*	Positive	0%	4
C1	ST15/KL19	*oqxA*, *oqxB*, *aac(3)-IId*, *aac(6′)Ib*, *aadA2*, *aph(3′)-Ia*, *strA*, *strB*, *bla*_CTX–M–15_, *bla*_DHA–1_, *bla*_KPC–2_, *bla*_OXA–1_, *bla*_SHV–28_, *bla*_TEM–1B_, *dfrA12*, *fosA*, *mph*(A), *sul1*, *sul2*	None	Negative	—	0
C29	ST15/KL19	*qnrB4*, *oqxA*, *oqxB*, *aac(3)-IId*, *aac(6′)Ib*, aadA2, *aph(3′)-Ia*, *armA*, *bla*_CTX–M–15_, *bla*_KPC–2_, *bla*_OXA–1_, *bla*_SHV–11_, *bla*_TEM–1B_, *catA2*, *fosA*, *mph*(A), *mph*(E), *msr*(E), *sul1*	*Yersiniabactin*, *aerobactin*, *rmpA2*	Negative	0%	4
C21	ST2237/KL19	*qnrB4*, *oqxA*, *oqxB*, *aph(3′)-Ia*, *armA*, *bla*_DHA–1_, *bla*_KPC–2_, *bla*_OXA–1_, *bla*_SHV–11_, *fosA*, *mph*(E), *msr*(E), *sul1*	*Yersiniabactin*, *aerobactin*, *rmpA2*	Negative	—	4
C17	ST2237/KL19	*qnrB4*, *oqxA*, *oqxB*, *aac(3)-IId*, *aadA2*, *aph(3′)-Ia*, *armA*, *bla*_DHA–1_, *bla*_KPC–2_, *bla*_SHV–11_, *dfrA12*, *fosA*, *mph*(A), *mph*(E), *msr*(E), *sul1*	*Yersiniabactin*, *aerobactin*, *rmpA2*	Negative	—	4
C2	ST2237/KL19	*qnrB4*, *oqxA*, *oqxB*, *aac(3)-IId*, *armA*, *bla*_DHA–1_, *bla*_KPC–2_, *bla*_SHV–11_, *fosA*, *mph*(E), *msr*(E), *sul1*	*Yersiniabactin*, *aerobactin*, *rmpA2*	Negative	—	4

*^*a*^The strain was not selected for *Galleria mellonella* larvae infection assay.*

*^*b*^Virulence score is determined by Kleborate software; virulence increases with the score.*

### Detailed Analysis of Novel *bla*_NDM–1_-Bearing Plasmids From Strain C20

**FIGURE 2:**
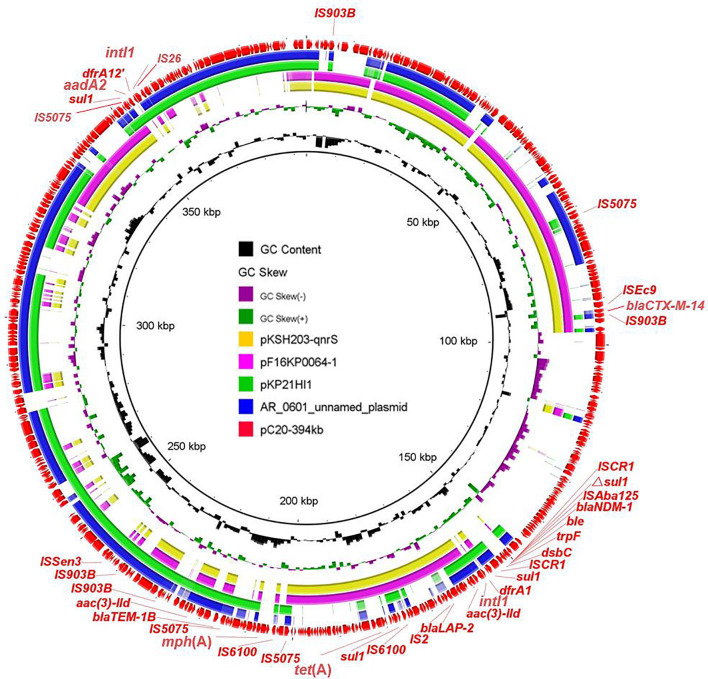
Circular comparison of pC20-394 kb and four most related plasmids available in NCBI. The outmost circle shows the coding genes of pC20-394 kb.

Five *bla*_NDM–1_-positive strains were separated to three clades based on phylogenetic analysis; therefore, three representative isolates from different branches were selected (C11, C20, and C39) for further exploration of genetic structures via MinION Nanopore long-read sequencing. The results showed that *bla*_NDM–1_ was located on three distinct plasmids IncX3, IncFIB/IncHI1B, and IncHI5-like, respectively. In strain C11, *bla*_NDM–1_ was found in typical IncX3 plasmid, which disseminated in human or animal sources worldwide and severed as the major vehicle of *bla*_NDM_ transmission to evolve with the generation of new NDM variants (Wu et al., 2019). In addition to *bla*_NDM–1_, the plasmid also carried *bla*_SHV–12_. In strain C20, *bla*_NDM–1_-bearing plasmid pC20-394 kb with 52.1% G + C content and 458 predicted ORF was 394 kb in size and possessed IncFIB and IncHI1B replicons. Apart from *bla*_NDM–1_, this latter plasmid harbored ESBL genes *bla*_CTX–M–14_, *bla*_LAP–2_, and *bla*_TEM–1B_; tetracycline resistance gene *tet*(A); aminoglycoside resistance genes *aadA2* and *aac(3)-IId*; sulfonamide resistance gene *sul1*; trimethoprim resistance genes *dfrA1* and *dfrA12*; and macrolide resistance gene *mph*(A). Except *bla*_CTX–M–14_, *aadA2*, and *dfrA12* genes, the remaining AMR genes were in a 97-kb MDR region. Despite that the plasmid could not transfer by conjugation, the coselection of *bla*_NDM–1_ may occur because of the existence of abundant AMR genes. Two integrons were found in different positions. The common genetic structure ΔIS*Aba125*-*bla*_NDM–1_-*ble*_MBL_-*trpF*-*dsbC* was embedded in downstream of In*183*, generating the complex class I integron In*183*-IS*CR1*-*bla*_NDM–1_ structure. Another In*1248*-like integron with the genetic array *intI1*-*dfrA12*-*aadA2*-*qacE*Δ*1*-*sul1* was flanked by IS*26* and IS*5075*. BLASTn search of pC20-394 kb against the NCBI nr database showed that less homologous sequences were found between this plasmid and the known plasmids; the maximum similarity was 99% identical at 58% coverage (pAR-0161_plasmid_unnamed, CP028952) (Figure 2 and Supplementary Figure 4). The emergence of novel *bla*_NDM–1_-bearing MDR plasmid in ST304 *K. pneumoniae* C20 implied that the novel plasmid mediated the transmission of *bla*_NDM–1_ and expanded the host ranges of *bla*_NDM–1_. Furthermore, the detailed analysis of IncHI5-like *bla*_NDM–1_-bearing plasmid in C39 has been reported in another study; the plasmid was 334,893 bp in length and possessed a large MDR region, which contained abundant AMR genes and mobile elements (Liu et al., 2021).

### Genetic Characteristics and Virulence Phenotype of Carbapenem-Resistant *Klebsiella pneumoniae* Indicate the Emergence of *bla*_KPC–2_-Positive Hypervirulent *Klebsiella pneumoniae*

**FIGURE 3:**
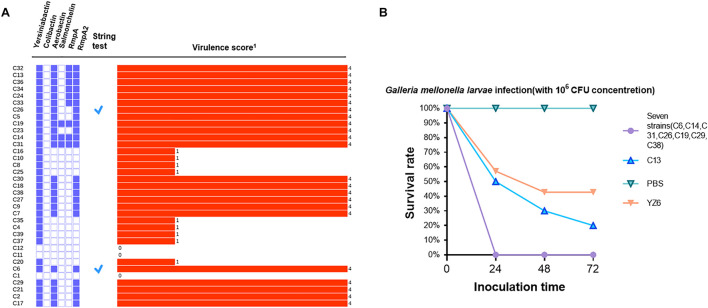
**(A)** Virulence gene distribution, string test, and virulence scores of all CRKP isolates. The distribution of virulence factors is shown in purple solid (positive) and hollow (negative) rectangles. A tick represents positive result of string test, and the virulence score is shown by a red bar and marked with corresponding numbers in right. **(B)**. Virulence potential of eight CRKP strains in a *G. mellonella* larvae infection model. The effect of 1 × 10^6^ CFU of each strain on survival was assessed in *G. mellonella* larvae. ^1^Virulence score is determined by Kleborate software; virulence increases with the score.

A total of six classes of virulence factor analysis were conducted among these isolates. Fewer virulence genes were possessed by *bla*_NDM–1_-positive strains than *bla*_KPC–2_-positive isolates. In *bla*_NDM–1_-positive strains, two isolates (C11 and C12) were not found to carry any virulence factors, whereas the remaining three isolates (C37, C39, and C20) harbored only one virulence factor yersiniabactin. Correspondingly, they also obtained lower virulence scores (Figure 3A). Analysis of the *ybt* locus revealed that 32 isolates were positive for the chromosomally encoded yersiniabactin, of which the dominant type was yersiniabactin lineage 9 within *ICEkp3* element distributed in all ST11-*bla*_KPC–2_ strains. Twenty-two *bla*_KPC–2_-positive strains harbored aerobactin lineage *iuc1* with aerobactin ST1. Besides, three salmochelin-producing strains, nine *rmpA*-positive strains, and 23 *rmpA2*-positive strains were also detected in *bla*_KPC–2_-positive strains. To get further insight into virulence phenotype of CRKP, all isolates were subjected to string test. The positive results were observed in C6 (ST15/KL24) and C26 (ST11/KL64). However, a negative string test could not predicate low virulence (Russo and Marr, 2019). Therefore, eight representative *bla*_KPC–2_-positive strains from clusters I and V, which contained all *bla*_KPC_-carrying strains, were conducted with *G. mellonella* larvae infection assay. Seven strains (C6, C14, C31, C26, C19, C29, and C38) resulted in 0% survival at 24 h with an inoculum of 10^6^ CFU, and the survival rate was 20% after the infection of C13 at 72 h (Figure 3B). No deaths were observed in PBS treatment group, and the survival rate of negative control YZ6 was higher than the experimental group.

### Comparative Analysis of Plasmids in ST11 *bla*_KPC–2_-Positive Strains

**FIGURE 4:**
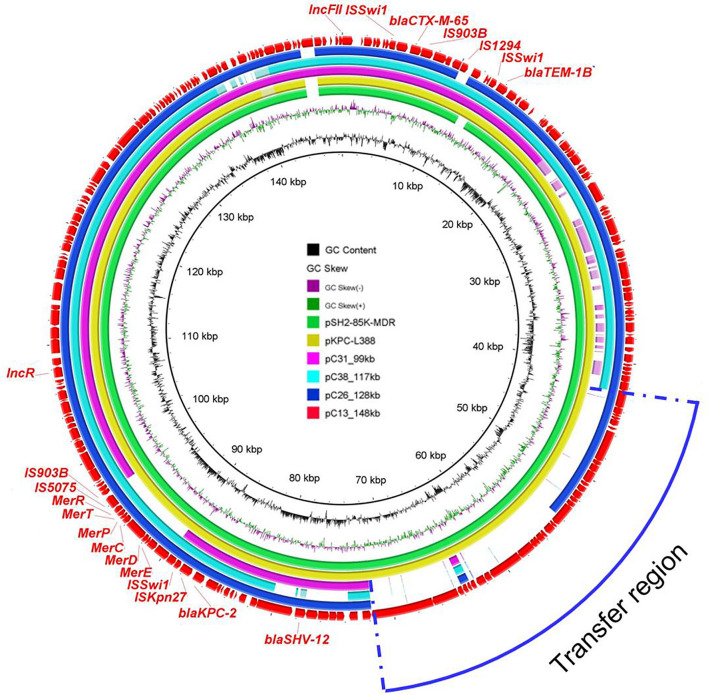
Circular comparison between the four *bla*_KPC–2_-bearing plasmids in this study and other most similar plasmids in the NCBI nr database. The plasmid pC13_148 kb was used as the reference in the outmost ring.

In order to gain further insights into the genetic basis of virulence and antibiotic resistance of plasmids harbored by ST11 *bla*_KPC–2_-positive isolates, plasmids of four ST11 strains (C13, C26, C31, and C38) were sequenced by MinION Nanopore sequencing platform. As the results showed, *bla*_KPC–2_-bearing plasmids of C13, C26, C31, and C38 shared similar backbone. These plasmids ranged from 99 to 148 kb and were classified as IncFII/IncR plasmid. Among them, the largest plasmid pC13_148 kb harbored by C13 was 148,462 bp and carried genes related to plasmid replicon, maintenance, conjugative elements, and AMR genes including *bla*_KPC–2_, *bla*_CTX–M–55_, *bla*_TEM–1B_, and *bla*_SHV–12_. BLASTn analysis demonstrated that it was similar to pSH2-85K-MDR (MH643792) and pKPC-L388 (CP029225) from *K. pneumoniae*, indicating the universal prevalence of this plasmid among *K. pneumoniae* (Figure 4). Besides, more detailed analysis of the remaining *bla*_KPC–2_-bearing plasmids in ST11 strains was performed using pC13_148 kb as reference. All ST11 isolates possessed this type of plasmid, with the absence of some specific regions. The *bla*_KPC–2_ gene was located on the same genetic context, flanked by genes belonging to the Tn*3*-based transposon family insertion sequences (IS*Kpn6* and IS*Kpn27*). Obviously, the deficiency of conjugation transfer region was observed in the majority of plasmids, which may explain why the *bla*_KPC–2_-bearing plasmids of most ST11 strains were non-conjugative. However, several *bla*_KPC–2_-bearing plasmids (C33, C32, C23, C19, and C14) with intact conjugation transfer regions were unable to be transferred successfully; the underlying mechanism warranted further study (Supplementary Figure 5).

**FIGURE 5:**
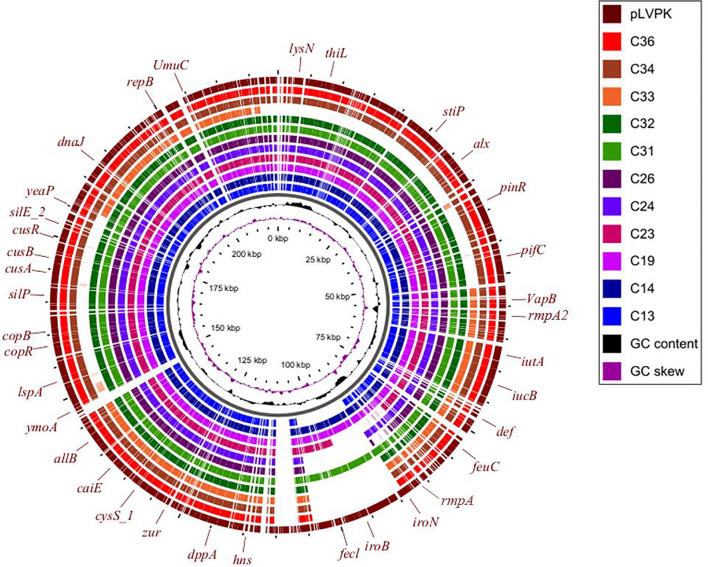
Circular comparison between the classical virulence plasmid pLVPK and the assembled plasmid contigs based on Illumina short-read data of the other 11 CRKP strains in this study.

Three virulence plasmids carried by C13, C26, and C31 were aligned well with classical virulence plasmid pLVPK (GenBank accession AY378100), a 219-kb plasmid that harbors *iroBCDN*, *iucABCD*, *rmpA*, and *rmpA2*. Furthermore, we found the similar plasmid structure presented in other eight ST11 strains based on Illumina-based contigs analysis (Figure 5).

### Phenotypic Assays Proved the Carbapenem-Resistant *Klebsiella pneumoniae* Isolates Had Remarkable Environmental Adaptability

To evaluate indicators of survival in the clinical settings of CRKP isolates in this study, according to the previous literature (Heiden et al., 2020), the fitness, desiccant resilience, biofilm formation, human serum resistance, and siderophore secretion assays were performed. To facilitate the analysis of the results, a total of 17 strains covering 15 representative CRKP isolates in this study and two control strains were divided into five groups, including A group (nine *bla*_KPC–2_-harboring ST11 strains), B (three *bla*_KPC–2_-harboring ST15 or ST2237 strains), C (three *bla*_NDM–1_-positive strains), D (*K. pneumoniae* ATCC700603), and E (clinical cKP HS11286 derivative YZ6).

**FIGURE 6:**
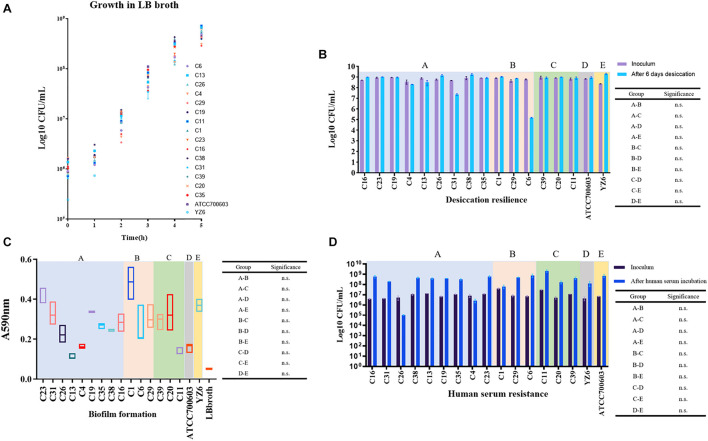
Results of phenotypic experiments to evaluate the fitness and viability of CRKP. Five groups with background color in pale purple (**A:** *bla*_KPC–2_-ST11 group), light pink (**B:** *bla*_KPC–2_-ST15 and ST2237 group), green (**C:** *bla*_NDM–1_-positive group), gray (**D:** standard strain ATCC700603), and yellow (**E:** YZ6), respectively. **(A)** Growth conditions of CRKP and control isolates over 5 h. **(B)** Comparison of the changes of CRKP and control isolates amount during desiccation. n.s., not significant. **(C)** Biofilm formation of CRKP and control isolates; the value represents the absorbance values at 590 nm. n.s., not significant. **(D)** Comparison of the changes of CRKP and control isolates amount incubation in human serum for 4 h. n.s., not significant.

We observed no significant difference in growth rates of the A, B, and C groups when compared to control groups. However, it was only found that the growth rate of the C group was significantly lower than that of group E at 2 h (*p* = 0.044 at 2 h) (Supplementary Table 7), which will be worth exploring further. Subsequently, we want to evaluate the performance of CRKP isolates under extreme dry environment, its capacity for biofilm formation, and the tolerance in human serum, which allowed us to assess the viability of CRKP in clinical settings and host. Desiccation resilience and human serum resistance experiments showed high survival rates of CRKP isolates under drying and human serum pressure. Comparable results were also obtained in biofilm formation, which suggested that these CRKP isolates will persist in this hospital for a long time, either under the clinical pressure or in patients (Figure 6).

**FIGURE 7:**
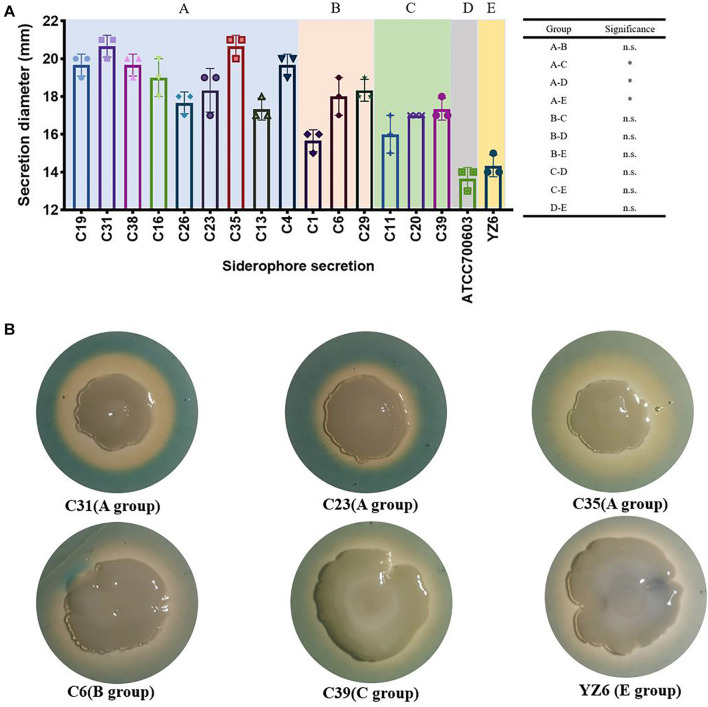
Results of siderophore secretion experiments. **(A)** The siderophore secretion diameters (showed in millimeters) of CRKP and control isolates. n.s., not significant, ^∗^*p* < 0.01. **(B)** The siderophore secretion zone of six representative isolates on CAS agar; the orange–yellow ring around the colonies indicates the siderophore secretion.

Nevertheless, the siderophore secretion capacity of A group (*bla*_KPC–2_-ST11) was significantly higher than C (*bla*_NDM–1_-positive group, *p* = 0.005), D (standard *K. pneumoniae* ATCC700603, *p* = 0.002), and E (YZ6, *p* = 0.005), but no significant difference was found between the A and B groups. This might be the role of the presence of yersiniabactin, aerobactin, and salmochelin in ST11 *bla*_KPC–2_-positive strains. However, the decreased siderophore secretion capacity was also observed in *bla*_KPC–2_-harboring ST15 strain C1, as it exhibited quite smaller secretion zone in absence of those genes encoding siderophores (Figure 7).

## Discussion

Our study systematically demonstrated the emergence of CRKP in a newly established hospital. Among these CRKP strains, we found *bla*_KPC–2_ harboring isolates with hypervirulence and multidrug resistance phenotype spread throughout the hospital for a long term, whereas *bla*_NDM–1_ carrying strains with novel ST types and plasmids were detected only in children cardiac ICU. Importantly, these isolates showed superior adaptive ability in clinical environment and host, which was likely due to the strong biofilm formation capacity. As a reservoir of pathogenic bacteria, hospital is often regarded as an ideal setting to investigate the epidemic characteristics of MDR strains (Yang et al., 2013; Wang et al., 2019), especially *K. pneumoniae* (Hu et al., 2020).

We identified the risk factors responsible for the occurrence of CRKP. Not surprisingly, it was found that exposure to carbapenem was associated with the emergence of CRKP, which was consistent with the previous investigations of CRKP (Liu et al., 2019a) and carbapenem-resistant *P. aeruginosa* (Lee et al., 2017; Zhang et al., 2018). Furthermore, previous study pointed out that carbapenem use with insufficient infection control measure might increase the risk of colistin resistance in *K. pneumoniae* (Gundogdu et al., 2018). Therefore, prudent carbapenem use is vital to reduce the production of drug-resistant bacteria in clinical settings.

It was found that the ST11 *bla*_KPC–2_-positive *K. pneumoniae* was the dominant strain in this hospital. However, the *bla*_KPC–2_-bearing plasmids among them were unable to transfer, which may due to the absence of the conjugation transfer genes in most plasmids. The genetic context of *bla*_KPC–2_ shared the core structure with IS*Kpn27*-*bla*_KPC–2_-IS*Kpn6*, suggesting that these mobile elements played a key role in the dissemination of *bla*_KPC–2_. Virulence assay revealed that most of *bla*_KPC–2_-positive strains were associated with hypervirulence, which could be mainly attributed to the existence of various virulence factors. Siderophore production was an important biomarker to distinguish hvKP and cKp (Russo et al., 2018). Generally, salmochelin, yersiniabactin, aerobactin, and enterobactin were regarded as typical siderophores to assist bacteria to acquire iron ion (Bachman et al., 2011) and involved in the virulence of Enterobacteriaceae and human infection (Schubert et al., 2000; Lam et al., 2018). The majority of *bla*_KPC–2_-positive *K. pneumoniae* encoded siderophore yersiniabactin and aerobactin, causing the siderophore secretion to be significantly higher than that of control groups. In addition, some strains carried the mucoid phenotype regulators *rmpA* and *rmpA2*, yet they were not positive for string test. It may be attributed to the fact that the expression of hypermucoviscosity phenotype was a fine-tuned process, which needed the mutual assistance of multiple genes (Walker et al., 2019). Moreover, the distribution of virulence factors in *bla*_KPC–2_ isolates may be diverse. For example, two virulence factors *rmpA2* and aerobactin, which had been detected in Illumina data of C38 strain, were not found in the complete plasmid sequence, manifesting that these two genes were located on chromosome. By contrast, the pLVPK-like plasmid was detected in 11 ST11 *bla*_KPC–2_ isolates. This plasmid harbored a set of virulence genes, including *iroBCDN*, *iucABCD*, *rmpA*, and *rmpA2*, indicating that the hypervirulent phenotypes of these strains were mediated by plasmids.

Unlike the traditional hypervirulent serotype KL1, KL2, and KL57, the major types of *bla*_KPC–2_-positive strains in this study were KL47 and KL64. In the early years, there were few reports regarding KL47 and KL64 hypervirulent *K. pneumoniae*. However, in recent 2 years, reports began to emerge (Liu et al., 2019b; Xie et al., 2020; Yang et al., 2020a; Zhang et al., 2020), and most of them were found in China. A recent study demonstrated that ST11-KL64 and ST11-KL47 isolates with enhanced virulence and transmissibility have emerged and undergone local expansion in China (Zhou et al., 2020). Our study also highlighted the potential hypervirulence of these two serotypes; more attention should be focused on them in further investigation.

In this study, *bla*_NDM–1_-positive CRKP occurred locally, as they were solely detected in children cardiac ICU with low virulence. The *bla*_NDM–1_ gene derived from pediatrics n China were frequently reported (Ding et al., 2019; Wang et al., 2020). *Klebsiella pneumoniae* was regarded as a key trafficker of AMR genes from environmental to clinical settings, and hundreds of mobile AMR genes have been found in this species (Wyres and Holt, 2018). Hence, it could explain why ST304 and ST1383 *K. pneumoniae* isolates were found to harbor *bla*_NDM_ in our study. Apart from IncX3 plasmid, *bla*_NDM_ was found in large MDR plasmids, including IncHI5-like and IncFIB/IncHI1B plasmids. The characteristics of IncFIB/IncHI1B plasmid harboring carbapenemase-encoding genes have been described previously (Matsumura et al., 2018). However, the structure of *bla*_NDM–1_-bearing plasmid containing IncFIB/IncHI1B replicons in our study was novel. It was a megaplasmid and carried the resistance determinants to heavy metals and several conjugal transfer genes. Abundant insertion sequences and two integrons were distributed in different locations among accessory regions, which might drive the formation of the novel structure of this plasmid. These findings alert us that the surveillance of *bla*_NDM–1_ in nosocomial setting needs to be strengthened.

There are still some drawbacks in this study. First, the sample size was not enough to objectively elucidate the distribution of CRKP in a large region. Second, the results of this study may not be able to apply to other hospitals. Besides, the strategy of sample collection should be improved in future studies; in addition to patients, more attention should focus on the nosocomial environment and staffs.

## Conclusion

The data presented in this study revealed two types of CRKP (*bla*_KPC–2_ and *bla*_NDM–1_) with distinct epidemiological features occurring in a newly established hospital in Henan province. Carbapenem exposure was associated with emergence of CRKP. These strains with superior viability constitute substantial threats in clinical settings. The clonal spread of ST11 hypervirulent *bla*_KPC–2_-positive *K. pneumoniae*, the occurrence of *bla*_NDM–1_-positive *K. pneumoniae* with novel ST type, and the dissemination of novel carbapenemase-encoding plasmids should be included in future surveillance priorities.

## Data Availability Statement

The datasets presented in this study can be found in online repositories. The names of the repository/repositories and accession number(s) can be found in the article/Supplementary Material.

## Author Contributions

RL and ZW designed and supervised the project. RC, ZL, PX, and XQ collected the strains, performed the experiments, and analyzed data. ZL, RL, and RC drafted the manuscript. RL and SQ revised the manuscript. All authors approved the final version for submission.

## Conflict of Interest

The authors declare that the research was conducted in the absence of any commercial or financial relationships that could be construed as a potential conflict of interest.

## Publisher’s Note

All claims expressed in this article are solely those of the authors and do not necessarily represent those of their affiliated organizations, or those of the publisher, the editors and the reviewers. Any product that may be evaluated in this article, or claim that may be made by its manufacturer, is not guaranteed or endorsed by the publisher.

## Footnotes

1
http://www.bldb.eu/BLDB.php?prot=A#KPC

2
http://www.carss.cn/Report/Details?aId=770

3
https://eucast.org/clinical_breakpoints/

4
https://cge.cbs.dtu.dk/services/

5
http://itol.embl.de/login.cgi

6
https://server.gview.ca/

7
http://rast.nmpdr.org/
