# Supplementary material for: Deciphering the Epidemiological Characteristics and Molecular Features of blaKPC–2- or blaNDM–1-Positive Klebsiella pneumoniae Isolates in a Newly Established Hospital
Source: Front Microbiol. 2021 Nov 10;12:741093. doi: 10.3389/fmicb.2021.741093 (PMC8631570; doi:10.3389/fmicb.2021.741093)
Supplement: Supplementary file 1 [file Data_Sheet_1.docx]

**Supplementary Data**

**Table S1. Summary of PCR primers and conditions in this study.**

| Primer | Sequence (5’-3’)^a^ | Gene | Production size (bp) | Group | PCR condition | Reference |
| --- | --- | --- | --- | --- | --- | --- |
| IMP-F | GGAATAGAGTGGCTTAAYTCTC | *bla*_IMP_ | 232 | Group I | 10 min at 94°C and 36 cycles of  amplification consisting of 30 s at 94°C, 40 s at 52°C, and  50 s at 72°C, with 5min at 72°C for the final extension. | (Poirel et al., 2011) |
| IMP-R | GGTTTAAYAAAACAACCACC |  |  |  |  |  |
| SPM-F | AAAATCTGGGTACGCAAACG | *bla*_SPM_ | 271 |  |  |  |
| SPM-R | ACATTATCCGCTGGAACAGG |  |  |  |  |  |
| VIM-F | GATGGTGTTTGGTCGCATA | *bla*_VIM_ | 390 |  |  |  |
| VIM-R | CGAATGCGCAGCACCAG |  |  |  |  |  |
| NDM-F | GGTTTGGCGATCTGGTTTTC | *bla*_NDM_ | 621 | Group II |  |  |
| NDM-R | CGGAATGGCTCATCACGATC |  |  |  |  |  |
| BIC-F | TATGCAGCTCCTTTAAGGGC | *bla*_BIC_ | 537 |  |  |  |
| BIC-R | TCATTGGCGGTGCCGTACAC |  |  |  |  |  |
| KPC-F | CGTCTAGTTCTGCTGTCTTG | *bla*_KPC_ | 798 |  |  |  |
| KPC-R | CTTGTCATCCTTGTTAGGCG |  |  |  |  |  |
| OXA-F | GCGTGGTTAAGGATGAACAC | *bla*_OXA-48_ | 438 |  |  |  |
| OXA-R | CATCAAGTTCAACCCAACCG |  |  |  |  |  |
| AIM-F | CTGAAGGTGTACGGAAACAC | *bla*_AIM_ | 390 | Group III |  |  |
| AIM-R | GTTCGGCCACCTCGAATTG |  |  |  |  |  |
| GIM-F | TCGACACACCTTGGTCTGAA | *bla*_GIM_ | 477 |  |  |  |
| GIM-R | AACTTCCAACTTTGCCATGC |  |  |  |  |  |
| SIM-F | TACAAGGGATTCGGCATCG | *bla*_SIM_ | 570 |  |  |  |
| SIM-R | TAATGGCCTGTTCCCATGTG |  |  |  |  |  |
| DIM-F | GCTTGTCTTCGCTTGCTAACG | *bla*_DIM_ | 699 |  |  |  |
| DIM-R | CGTTCGGCTGGATTGATTTG |  |  |  |  |  |
| Khe-F3 | CCGGAGCGTTTTTCAATCGGCG | *khe* | 441 | Group IV | 94°C for 10 min, followed by 35 cycles at 94°C for 30 s, 60°C for 30 s, 72°C  for 60 s, and a final extension step of 72°C for 5 min. | (Yu et al., 2018) |
| Khe-R3 | CGCTTCGCCCCTCACCTGAAAT |  |  |  |  |  |
| pilV-F3 | CGATGGCGCTGGCGACGATTAT | *pilV* | 627 |  |  |  |
| pilV-R3 | CCCGATGGGCAAGAACATGCGT |  |  |  |  |  |
| kphp-F3 | TGGCGGGTAATGCCCGATCAGT | *kphp* | 236 |  |  |  |
| kphp-R3 | AGGCCGCTTTCCATAAGCCGTT |  |  |  |  |  |
| pilL-F2 | CGGTATTTGCTCTGCGTGATAG | *pill* | 350 |  |  |  |
| pilL-R2 | TGGTTATACAGAACGGCATTGG |  |  |  |  |  |
| ST65-F1 | GCTATGCCAATGCCGGAACCGT | Specific target sequences for ST65/ST375 | 146 |  |  |  |
| ST65-R1 | ACCCACCCCCGTAAAGATGGCA |  |  |  |  |  |
| ST23-F1 | ACGCATGCCTACAGGACATCGAA | Specific target sequences for ST23 | 518 |  |  |  |
| ST23-R1 | TCCAGTAGGAGCAGACAGCGGA |  |  |  |  |  |
| ST86-F2 | TATTCTTTCTAGGGGCCGAATG | Specific target sequences for ST86 | 778 |  |  |  |
| ST86-R2 | TCACCAGCTCACTTGAACATTA |  |  |  |  |  |
| wzyK1-F1 | ACGGAGCAATATGGCCAGTCCG | *wzy_K1_* | 174 | Group V |  |  |
| wzyK1-R1 | GCCAACAATTCCCGTTTCTGCTGC |  |  |  |  |  |
| wzyK2-F1 | TATTCGAGCTGGCGCCGCCTTA | *wzy_K2_* | 451 |  |  |  |
| wzyK2-R1 | CGTTCCCCTTGTTGCCACGGAT |  |  |  |  |  |
| wzyKL64-F1 | TCAGTTCCGACCCTGATGCAGGTA | *wzy_KL64_* | 268 |  |  |  |
| wzyKL64-R1 | GCCAGAGCAACTATCATCCAAAGCCA |  |  |  |  |  |
| wzyKL47-F1 | GGACGCACAGTTTCCCAATTCGC | *wzy_KL47_* | 392 |  |  |  |
| wzyKL47-R1 | GCCCACATGAACCCACTTGGCA |  |  |  |  |  |
| rmpA-F1 | ATGTGGCTTGACGTTTCGGGGG | *rmpA* | 160 | Group VI |  |  |
| rmpA-R1 | GCCGTGGATAATGGTTTACAATTCGGC |  |  |  |  |  |
| rmpA2-F1 | GGATGTGGCTTGACATTTCGGGGG | *rmpA2* | 227 |  |  |  |
| rmpA2-R1 | TTCATGGATGCCCTCCCTCCTG |  |  |  |  |  |
| HI1B-F1 | TCGCTACTGCGATTGGGGGTCT | IncHI1B replicon | 351 |  |  |  |
| HI1B-R1 | GAAATGGGTGTGCTGGAGCCGT |  |  |  |  |  |
| iroN-F1 | CCGCAAAGAGACGAACCGCCTT | *iroN* | 546 |  |  |  |
| iroN-R1 | CGGGCAATCCCCGCTTTGACTT |  |  |  |  |  |
| iutA-F1 | AATCACCTGGGGGCTGGATGCT | *iutA* | 683 |  |  |  |
| iutA-R1 | CCGCACCTTCCACGCCGTAAAT |  |  |  |  |  |

^a^ D = A or G or T; Y = C or T.

**Table S2. Statistics of isolates during March 2018 to August 2019**

| Species | Number of isolates | Proportion (%) |
| --- | --- | --- |
| *Klebsiella pneumoniae* | 193 | 14 |
| *Acinetobacter baumannii* | 177 | 13 |
| *Pseudomonas aeruginosa* | 158 | 11 |
| *Escherichia coli* | 116 | 8 |
| *Staphylococcus aureus* | 92 | 7 |
| *Stenotrophomonas maltophilia* | 78 | 6 |
| *Burkholderia cepacia* | 57 | 4 |
| *Enterobacter cloacae* | 51 | 4 |
| *Streptococcus pneumoniae* | 44 | 3 |
| *Haemophilus influenzae* | 41 | 3 |
| *Corynebacterium striatum* | 41 | 3 |
| *Staphylococcus epidermidis* | 39 | 3 |
| *Serratia marcescens* | 27 | 2 |
| *Ralstonia pickettii* | 23 | 2 |
| *Staphylococcus haemolyticus* | 23 | 2 |
| *Enterococcus faecium* | 23 | 2 |
| *Enterobacter aerogenes* | 21 | 1 |
| Other | 209 | 14 |
| Total^a^ | 1413 |  |

*^a^* Including 377 gram-positive and 1036 gram-negative stains

**Table S3. Position statistics of *K. pneumoniae* and CRKP**

|  | Coronary care unit | Thoracic surgery ward | Respiratory medicine ward | Comprehensive intensive care unit | Children cardiac intensive care unit | Adult cardiac intensive care unit | Nephrology ward | Coronary heart disease ward III | Heart failure ward | Cardiovascular surgery intensive care unit | Coronary heart disease ward II | Adult cardiac surgery IV | Adult cardiac surgery I | Orthopedic ward | Neurological disease ward | Digestive disease ward | Coronary heart disease ward I | Adult cardiac surgery III | Total |
| --- | --- | --- | --- | --- | --- | --- | --- | --- | --- | --- | --- | --- | --- | --- | --- | --- | --- | --- | --- |
| No. of *K. pneumoniae* | 14 | 5 | 16 | 21 | 25 | 84 | 3 | 2 | 3 | 5 | 1 | 2 | 2 | 4 | 2 | 2 | 1 | 1 | 193 |
| No. of CRKP | 5 | 3 | 7 | 7 | 7 | 2 | 1 | 1 | 1 | 0 | 0 | 0 | 0 | 0 | 1 | 0 | 0 | 0 | 35 |

**Table S4. MICs (mg/L) of all CRKP strains in this study.**

|  | CIP | LVX | CHL | ATM | AMP | SAM | PIP | TZP | AMC | GEN | AMK | CZO | CAZ | CTX | FEP | MEM | IPM | SXT | POL | TGC^1^ | TCY |
| --- | --- | --- | --- | --- | --- | --- | --- | --- | --- | --- | --- | --- | --- | --- | --- | --- | --- | --- | --- | --- | --- |
| C1 | >2 | 8 | ≤4 | >16 | >16 | >16 | >64 | >64 | >16 | >8 | ≤8 | >16 | >16 | >32 | >16 | >8 | >8 | >2 | ≤0.5 | 19 | >8 |
| C2 | >2 | >8 | >16 | >16 | >16 | >16 | >64 | >64 | >16 | >8 | >32 | >16 | >16 | 32 | >16 | >8 | >8 | ≤0.5 | ≤0.5 | 13 | >8 |
| C4 | >2 | >8 | >16 | >16 | >16 | >16 | >64 | >64 | >16 | >8 | ≤8 | >16 | 16 | >32 | >16 | 8 | >8 | >2 | 1 | 19 | >8 |
| C5 | >2 | >8 | >16 | >16 | >16 | >16 | >64 | >64 | >16 | >8 | >32 | >16 | >16 | >32 | >16 | >8 | >8 | ≤0.5 | ≤0.5 | 19 | 4 |
| C6 | >2 | >8 | >16 | >16 | >16 | >16 | >64 | >64 | >16 | >8 | >32 | >16 | >16 | 16 | 16 | 8 | >8 | >2 | ≤0.5 | 21 | 4 |
| C7 | >2 | >8 | 8 | >16 | >16 | >16 | >64 | >64 | >16 | >8 | >32 | >16 | >16 | >32 | >16 | >8 | >8 | ≤0.5 | ≤0.5 | 19 | >8 |
| C8 | >2 | >8 | >16 | >16 | >16 | >16 | >64 | >64 | >16 | >8 | >32 | >16 | >16 | >32 | >16 | >8 | >8 | ≤0.5 | ≤0.5 | 18 | 4 |
| C9 | >2 | >8 | 8 | >16 | >16 | >16 | >64 | >64 | >16 | >8 | >32 | >16 | >16 | >32 | >16 | >8 | >8 | ≤0.5 | ≤0.5 | 18 | >8 |
| C10 | >2 | >8 | >16 | >16 | >16 | >16 | >64 | >64 | >16 | >8 | >32 | >16 | >16 | >32 | >16 | >8 | >8 | >2 | 1 | 18 | >8 |
| C11 | ≤0.5 | ≤1 | 8 | >16 | >16 | >16 | >64 | >64 | >16 | ≤2 | ≤8 | >16 | >16 | >32 | >16 | >8 | >8 | ≤0.5 | 1 | 18 | ≤2 |
| C12 | ≤0.5 | ≤1 | 8 | >16 | >16 | >16 | >64 | >64 | >16 | ≤2 | ≤8 | >16 | >16 | >32 | >16 | >8 | >8 | ≤0.5 | 1 | 19 | 4 |
| C13 | >2 | >8 | >16 | >16 | >16 | >16 | >64 | >64 | >16 | >8 | >32 | >16 | >16 | >32 | >16 | >8 | >8 | >2 | ≤0.5 | 19 | >8 |
| C14 | >2 | >8 | 8 | >16 | >16 | >16 | >64 | >64 | >16 | >8 | >32 | >16 | >16 | >32 | >16 | >8 | >8 | ≤0.5 | ≤0.5 | 21 | 4 |
| C16 | >2 | >8 | 8 | >16 | >16 | >16 | >64 | >64 | >16 | >8 | ≤8 | >16 | >16 | >32 | >16 | >8 | >8 | >2 | ≤0.5 | 19 | >8 |
| C17 | >2 | >8 | 8 | >16 | >16 | >16 | >64 | >64 | >16 | >8 | >32 | >16 | >16 | >32 | >16 | >8 | >8 | >2 | ≤0.5 | 20 | 4 |
| C18 | >2 | >8 | >16 | >16 | >16 | >16 | >64 | >64 | >16 | >8 | >32 | >16 | >16 | >32 | >16 | >8 | >8 | ≤0.5 | 1 | 20 | 4 |
| C19 | >2 | >8 | 8 | >16 | >16 | >16 | >64 | >64 | >16 | >8 | >32 | >16 | >16 | >32 | >16 | >8 | >8 | ≤0.5 | 1 | 19 | 4 |
| C20 | 1 | ≤1 | 8 | >16 | >16 | >16 | >64 | >64 | >16 | >8 | ≤8 | >16 | >16 | >32 | >16 | >8 | >8 | >2 | 1 | 18 | >8 |
| C21 | >2 | >8 | >16 | >16 | >16 | >16 | >64 | >64 | >16 | >8 | >32 | >16 | >16 | >32 | >16 | >8 | >8 | ≤0.5 | 1 | 18 | >8 |
| C23 | >2 | >8 | 8 | >16 | >16 | >16 | >64 | >64 | >16 | >8 | >32 | >16 | >16 | >32 | >16 | >8 | >8 | >2 | ≤0.5 | 20 | 4 |
| C24 | >2 | >8 | 8 | >16 | >16 | >16 | >64 | >64 | >16 | >8 | >32 | >16 | >16 | >32 | >16 | >8 | >8 | >2 | ≤0.5 | 19 | >8 |
| C25 | >2 | >8 | 8 | >16 | >16 | >16 | >64 | >64 | >16 | ≤2 | ≤8 | >16 | >16 | >32 | >16 | >8 | >8 | ≤0.5 | 1 | 19 | 4 |
| C26 | >2 | >8 | >16 | >16 | >16 | >16 | >64 | >64 | >16 | >8 | >32 | >16 | >16 | >32 | >16 | 8 | >8 | >2 | 1 | 19 | >8 |
| C27 | >2 | >8 | 8 | >16 | >16 | >16 | >64 | >64 | >16 | >8 | >32 | >16 | >16 | >32 | >16 | >8 | >8 | >2 | ≤0.5 | 20 | 4 |
| C29 | >2 | 8 | >16 | >16 | >16 | >16 | >64 | >64 | >16 | >8 | >32 | >16 | >16 | >32 | >16 | >8 | >8 | >2 | ≤0.5 | 19 | >8 |
| C30 | >2 | >8 | >16 | >16 | >16 | >16 | >64 | >64 | >16 | >8 | >32 | >16 | >16 | >32 | >16 | >8 | >8 | ≤0.5 | ≤0.5 | 20 | 4 |
| C31 | >2 | >8 | >16 | >16 | >16 | >16 | >64 | >64 | >16 | >8 | >32 | >16 | >16 | >32 | >16 | >8 | >8 | >2 | 1 | 19 | >8 |
| C32 | >2 | >8 | >16 | >16 | >16 | >16 | >64 | >64 | >16 | >8 | >32 | >16 | >16 | >32 | >16 | >8 | >8 | ≤0.5 | ≤0.5 | 19 | >8 |
| C33 | >2 | >8 | 8 | >16 | >16 | >16 | >64 | >64 | >16 | >8 | >32 | >16 | >16 | >32 | >16 | >8 | >8 | >2 | ≤0.5 | 19 | >8 |
| C34 | >2 | >8 | 8 | >16 | >16 | >16 | >64 | >64 | >16 | >8 | >32 | >16 | >16 | >32 | >16 | >8 | >8 | >2 | ≤0.5 | 19 | >8 |
| C35 | >2 | >8 | 8 | >16 | >16 | >16 | >64 | >64 | >16 | >8 | ≤8 | >16 | >16 | >32 | >16 | >8 | 8 | ≤0.5 | ≤0.5 | 19 | >8 |
| C36 | >2 | >8 | 8 | >16 | >16 | >16 | >64 | >64 | >16 | >8 | >32 | >16 | >16 | >32 | >16 | >8 | >8 | >2 | ≤0.5 | 19 | >8 |
| C37 | ≤0.5 | ≤1 | ≤4 | >16 | >16 | >16 | >64 | >64 | >16 | >8 | ≤8 | >16 | >16 | >32 | >16 | >8 | >8 | >2 | ≤0.5 | 20 | 2 |
| C38 | >2 | >8 | 8 | >16 | >16 | >16 | >64 | >64 | >16 | >8 | >32 | >16 | >16 | >32 | >16 | >8 | >8 | >2 | ≤0.5 | 20 | >8 |
| C39 | ≤0.5 | ≤1 | ≤4 | >16 | >16 | >16 | >64 | >64 | >16 | >8 | ≤8 | >16 | >16 | >32 | >16 | >8 | >8 | >2 | ≤0.5 | 20 | 2 |

^1^ The MICs of tigecycline were determined by disk diffusion method.

CIP Ciprofloxacin, LVX Levofloxacin, CHL Chloramphenicol, ATM Aztreonam, AMP Ampicillin, SAM Ampicillin-Sulbactam, PIP Piperacillin, TZP Piperacillin-Tazobactam, AMC Amoxicillin-Clavulanic acid, GEN Gentamicin, AMK Amikacin, CZO Cefazolin, CAZ Ceftazidime, CTX Cefotaxime, FEP Cefepime, MEM Meropenem, IPM Imipenem, SXT Trimethoprim-Sulfamethoxazole, POL Polymyxin B, TGC Tigecycline, TCY Tetracycline.

**Table S5. Analysis of risk factors related to 35 CRKP strains.**

|  | Value for group | |  |  |
| --- | --- | --- | --- | --- |
| Variable | CRKP positive | CRKP negative | P-value | OR (95%CI)^a^ |
| No. of patients (%) | 35 (18%) | 158 (82%) |  |  |
| No. of male patients (%) | 23 (66%) | 109 (69%) | 0.871 | 1.083 (0.413-2.838) |
| No. of ≥50 years old patients (%) | 25 (71%) | 115 (73%) | 0.322 | 0.610 (0.230-1.623) |
| No. of receiving carbapenem treatment (%)^b^ | 25 (71%) | 68 (43%) | 0.009 | 3.453 (1.369-8.707) |
| No. of ICU patients (%) | 21 (60%) | 128 (81%) | 0.252 | 0.515 (0.165-1.602) |
| No. of isolation season with |  |  |  |  |
| During January to March | 11 (31%) | 41 (26%) | NA^c^ | 1 |
| During April to June | 15 (43%) | 36 (23%) | 0.469 | 1.469 (0.519-4.164) |
| During July to September | 5 (14%) | 45 (28%) | 0.097 | 0.334 (0.092-1.220) |
| During October to December | 4 (11%) | 36 (23%) | 0.367 | 0.536 (0.138-2.080) |
| No. of sample type |  |  |  |  |
| Blood | 4 (11%) | 13 (8%) | NA | 1 |
| Ascitic fluid | 2 (6%) | 1 (0.6%) | 0.127 | 8.911 (0.537-147.754) |
| Sputum | 17 (49%) | 126 (80%) | 0.482 | 0.610 (0.154-2.422) |
| Bronchoalveolar Fluid | 4 (11%) | 6 (4%) | 0.705 | 1.439 (0.219-9.451) |
| Wound secretion | 3 (8.5%) | 4 (2.5%) | 0.404 | 2.509 (0.289-21.780) |
| Urine | 4 (11%) | 6 (4%) | 0.355 | 2.510 (0.357-17.619) |
| Ductus venosus | 1 (3%) | 2 (1%) | 0.579 | 2.206 (0.135-36.079) |

^a^ OR= odds ratio; CI= confidence interval.

^b^ Shading indicated risk factors with statistically significant (P-value <0.05).

^c^ NA= not available.

**Table S6. Clinical information of patients infected with CRKP.**

| Strain number | Isolation date | Sample | Ward | Gender | Age | Diagnose | Medication history |
| --- | --- | --- | --- | --- | --- | --- | --- |
| C1 | 2018/3/13 | Blood | Coronary care unit (CCU) | Male | 60-65 | Arhythmia, Cerebral infraction, Diabetes | Meropenem, Cefoperazone/sulbactam, |
| C2 | 2018/3/20 | Ascitic fluid | Thoracic surgery ward (TS) | Male | 70-75 | Esophagus cancer, Diabetes | Tigecycline, Cefoperazone/sulbactam |
| C4 | 2018/3/26 | Sputum | Respiratory medicine ward (RM) | Male | 60-65 | Pulmonary infection, Spinal cord injury | Cefoperazone/sulbactam, Tigecycline, Amikacin |
| C5 | 2018/3/26 | Sputum | Comprehensive intensive care unit (CICU) | Male | 65-70 | Pulmonary infection | Piperacillin/tazobactam, Linezolid, Meropenem, Tigecycline |
| C6 | 2018/3/29 | Wound Secretion | Thoracic surgery ward (TS) | Female | 60-65 | Pulmonary infection, hypertension | Cefoxitin, Cefoperazone/sulbactam, Meropenem, Tigecycline, Levofloxacin |
| C7 | 2018/4/4 | Sputum | Neurology ward (NEU) | Male | 50-55 | Brainstem hemorrhage, Pulmonary infection | Tigecycline, Cotrimoxazole, Cefoperazone/sulbactam |
| C8 | 2018/5/12 | Ascitic fluid | Comprehensive intensive care unit (CICU) | Female | 60-65 | Multiple organ failure | Levofloxacin, Cefoperazone/sulbactam, Meropenem, Tigecycline, Imipenem |
| C9 | 2018/5/19 | Sputum | Children cardiac intensive care unit (CCICU) | Male | 0-5 | Congenital heart disease | Meropenem, Cefoperazone/sulbactam, Cefuroxime, Cotrimoxazole |
| C10 | 2018/5/20 | Sputum | Comprehensive intensive care unit (CICU) | Male | 65-70 | Pulmonary infection, Coronary atherosclerosis | Imipenem, Tigecycline |
| C11 | 2018/5/31 | Sputum | Children cardiac intensive care unit (CCICU) | Female | 0-5 | Congenital heart disease | Linezolid, Cefuroxime, Imipenem, Meropenem, Cotrimoxazole |
| C12 | 2018/6/1 | Sputum | Children cardiac intensive care unit (CCICU) | Male | 0-5 | Pneumoniae | Cefuroxime, Cotrimoxazole, Vancomycin, Meropenem, Linezolid |
| C13 | 2018/6/7 | Bronchoalveolar Fluid | Respiratory medicine ward (RM) | Male | 70-75 | Pneumoniae | Cefuroxime, Levofloxacin, Piperacillin/tazobactam |
| C14 | 2018/6/13 | Bronchoalveolar Fluid | Respiratory medicine ward (RM) | Male | 50-55 | Pulmonary infection | Cefoperazone/sulbactam, Levofloxacin, Ceftazidime, Imipenem, Tigecycline, Meropenem |
| C16 | 2018/8/21 | Wound secretion | Thoracic surgery ward (TS) | Female | 30-35 | Pneumoniae, severe acute pancreatitis | Tigecycline, Meropenem, Linezolid, Piperacillin/tazobactam |
| C17 | 2018/8/23 | Wound Secretion | Children cardiac intensive care unit (CCICU) | Male | 15-20 | Congenital heart disease | None |
| C18 | 2018/10/19 | Sputum | Adult cardiac intensive care unit (ACICU) | Female | 65-70 | Pulmonary infection, Coronary atherosclerosis | Cefuroxime, Meropenem, Tigecycline, Teicoplanin |
| C19 | 2018/10/27 | Blood | Comprehensive intensive care unit (CICU) | Male | 65-70 | Multiple organ dysfunction syndrome | Meropenem, Piperacillin/tazobactam, Linezolid |
| C20 | 2018/11/14 | Sputum | Children cardiac intensive care unit (CCICU) | Female | 0-5 | Congenital heart disease | Azithromycin, Meropenem, Piperacillin/tazobactam |
| C21 | 2018/12/24 | Blood | Comprehensive intensive care unit (CICU) | Male | 75-80 | Respiratory failure, Pulmonary infection | Meropenem, Tigecycline, Cefoperazone/sulbactam |
| C23 | 2019/1/4 | Sputum | Comprehensive intensive care unit (CICU) | Male | 55-60 | - Malignant tumor in upper lobe of right lung | Cefuroxime, Cefoperazone/sulbactam, Levofloxacin, Teicoplanin, Meropenem, Tigecycline |
| C24 | 2019/1/11 | Urine | Coronary care unit (CCU) | Male | 60-65 | Coronary heart disease | Cefoperazone/sulbactam, Teicoplanin, Meropenem, Tigecycline, Piperacillin/tazobactam, Linezolid, Ceftriaxone |
| C25 | 2019/1/28 | Sputum | Respiratory medicine ward (RM) | Male | 70-75 | Pulmonary infection | Levofloxacin, Tigecycline, Amikacin, Cefoperazone/sulbactam, Ceftazime |
| C26 | 2019/2/28 | Blood | Comprehensive intensive care unit (CICU) | Female | 60-65 | Pulmonary infection, Respiratory failure | Meropenem, Tigecycline |
| C27 | 2019/3/11 | Bronchoalveolar Fluid | Respiratory medicine ward (RM) | Male | 80-85 | Pneumoniae, respiratory failure | Levofloxacin, Cotrimoxazole, Meropenem, Tigecycline |
| C29 | 2019/3/20 | Sputum | Coronary care unit (CCU) | Male | 65-70 | Coronary heart disease | Imipenem, Tigecycline |
| C30 | 2019/4/25 | Sputum | Adult cardiac intensive care unit (ACICU) | Female | 55-60 | Coronary heart disease | Levofloxacin, Ceftazidime, Teicoplanin |
| C31 | 2019/5/9 | Urine | Nephrology ward (NEP) | Female | 80-85 | Chronic renal failure | Tigecycline, Imipenem, Cefoperazone/sulbactam |
| C32 | 2019/5/18 | Ductus venosus | Coronary heart disease ward (CHD) | Male | 55-60 | Coronary heart disease | Tigecycline, Cefoxitin |
| C33 | 2019/6/3 | Urine | Respiratory medicine ward (RM) | Female | 30-35 | Epilepsy, Pulmonary infection | None |
| C34 | 2019/6/20 | Sputum | Coronary care unit (CCU) | Male | 55-60 | Coronary heart disease | Imipenem, Teicoplanin, Tigecycline, Meropenem |
| C35 | 2019/6/24 | Sputum | Respiratory medicine ward (RM) | Male | 70-75 | Pulmonary infection | Cefoperazone/sulbactam, Teicoplanin, Meropenem, Tigecycline, Piperacillin/tazobactam, Linezolid, Imipenem |
| C36 | 2019/6/24 | Bronchoalveolar Fluid | Coronary care unit (CCU) | Male | 10-15 | Myocarditis | Cefoperazone/sulbactam, Teicoplanin, Meropenem, Tigecycline, Piperacillin/tazobactam, Linezolid, Imipenem, Vancomycin |
| C37 | 2019/7/31 | Sputum | Children cardiac intensive care unit (CCICU) | Female | 0-5 | Congenital heart disease | Cefoperazone/sulbactam, Meropenem, Cotrimoxazole |
| C38 | 2019/7/18 | Urine | Heart failure ward (HF) | Female | 60-65 | Heart failure | Cefoperazone/sulbactam |
| C39 | 2019/8/10 | Sputum | Children cardiac intensive care unit (CCICU) | Male | 0-5 | Congenital heart disease | Imipenem |

**Table S7. The growth rates of 15 CRKP isolates and 2 control strains in continuous 5 hours (calculated every hour).**

|  | C13 | C26 | C4 | C19 | C23 | C16 | C38 | C31 | C35 | C6 | C29 | C1 | C11 | C39 | C20 | ATCC700603 | YZ6 |
| --- | --- | --- | --- | --- | --- | --- | --- | --- | --- | --- | --- | --- | --- | --- | --- | --- | --- |
| 1h | 1.04±0.0087 | 1.04±0.009 | 1.06±0.004 | 1.01±0.001 | 1.02±0.008 | 1.01±0.009 | 1.07±0.0004 | 1.06±0.012 | 1.03±0.003 | 1.02±0.002 | 1.04±0.003 | 1.07±0.005 | 1.04±0.002 | 1.06±0.013 | 1.07±0.011 | 1.05±0.022 | 1.09±0.0003 |
| 2h | 1.11±0.0032 | 1.12±0.003 | 1.06±0.01 | 1.10±0.01 | 1.12±0.004 | 1.10±0.007 | 1.11±0.0004 | 1.14±0.0005 | 1.13±0.006 | 1.11±0.002 | 1.06±0.001 | 1.11±0.011 | 1.14±0.005 | 1.12±0.007 | 1.14±0.017 | 1.14±0.009 | 1.15±0.001 |
| 3h | 1.10±0.018 | 1.12±0.014 | 1.15±0.024 | 1.13±0.019 | 1.10±0.008 | 1.13±0.015 | 1.11±0.00007 | 1.08±0.008 | 1.13±0.008 | 1.13±0.022 | 1.17±0.006 | 1.12±0.002 | 1.14±0.001 | 1.14±0.007 | 1.11±0.005 | 1.13±0.008 | 1.11±0.012 |
| 4h | 1.10±0.016 | 1.04±0.012 | 1.06±0.017 | 1.09±0.014 | 1.05±0.015 | 1.09±0.015 | 1.09±0.016 | 1.08±0.004 | 1.06±0.010 | 1.08±0.010 | 1.08±0.001 | 1.09±0.027 | 1.08±0.007 | 1.09±0.012 | 1.07±0.004 | 1.06±0.011 | 1.08±0.012 |
| 5h | 1.04±0.002 | 1.08±0.008 | 1.04±0.006 | 1.01±0.011 | 1.04±0.022 | 1.03±0.001 | 1.00±0.003 | 1.06±0.005 | 1.04±0.006 | 1.06±0.003 | 1.04±0.002 | 1.01±0.010 | 1.04±0.003 | 1.01±0.004 | 1.04±0.004 | 1.04±0.011 | 1.07±0.009 |

Note: Five kinds of color mean five different groups, consistent with Figure 6.


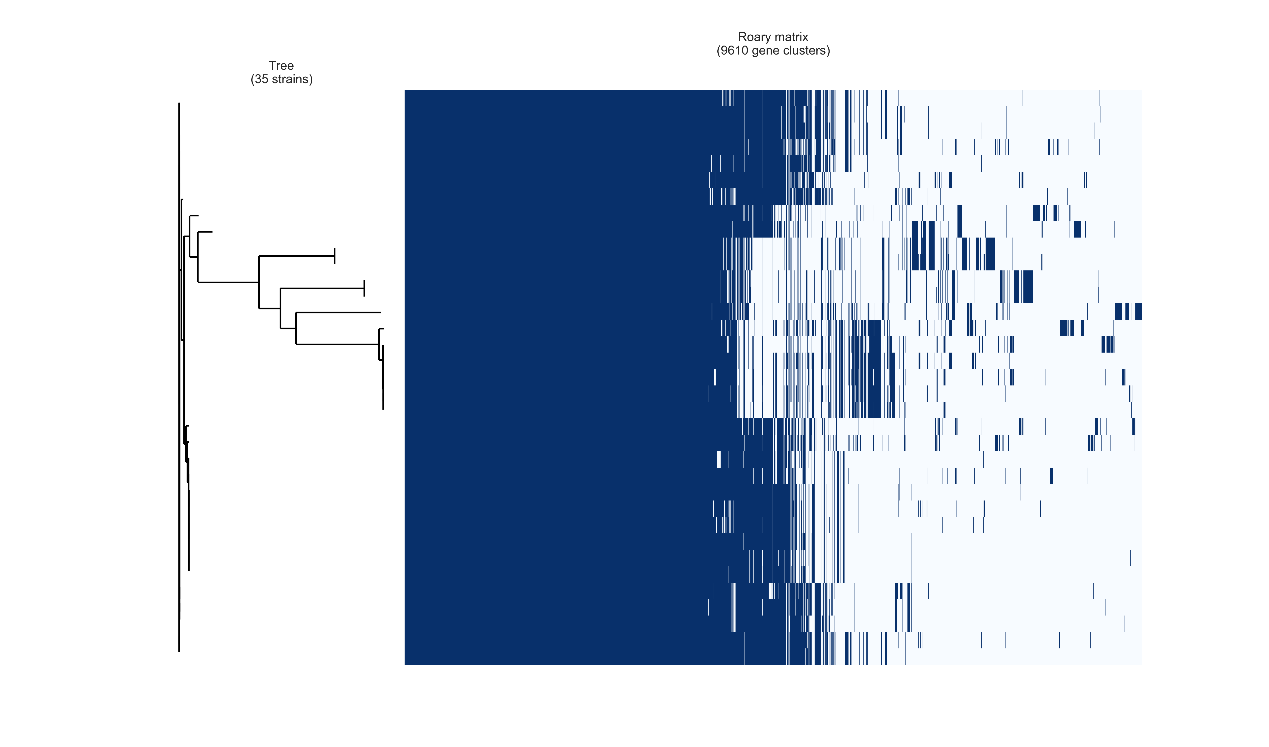


**Figure S1. Pangenome analysis of thirty-five CRKP strains revealed by Roary.** The blue bar indicated the pangenome of CRKP, including the 9610 annotated genes.


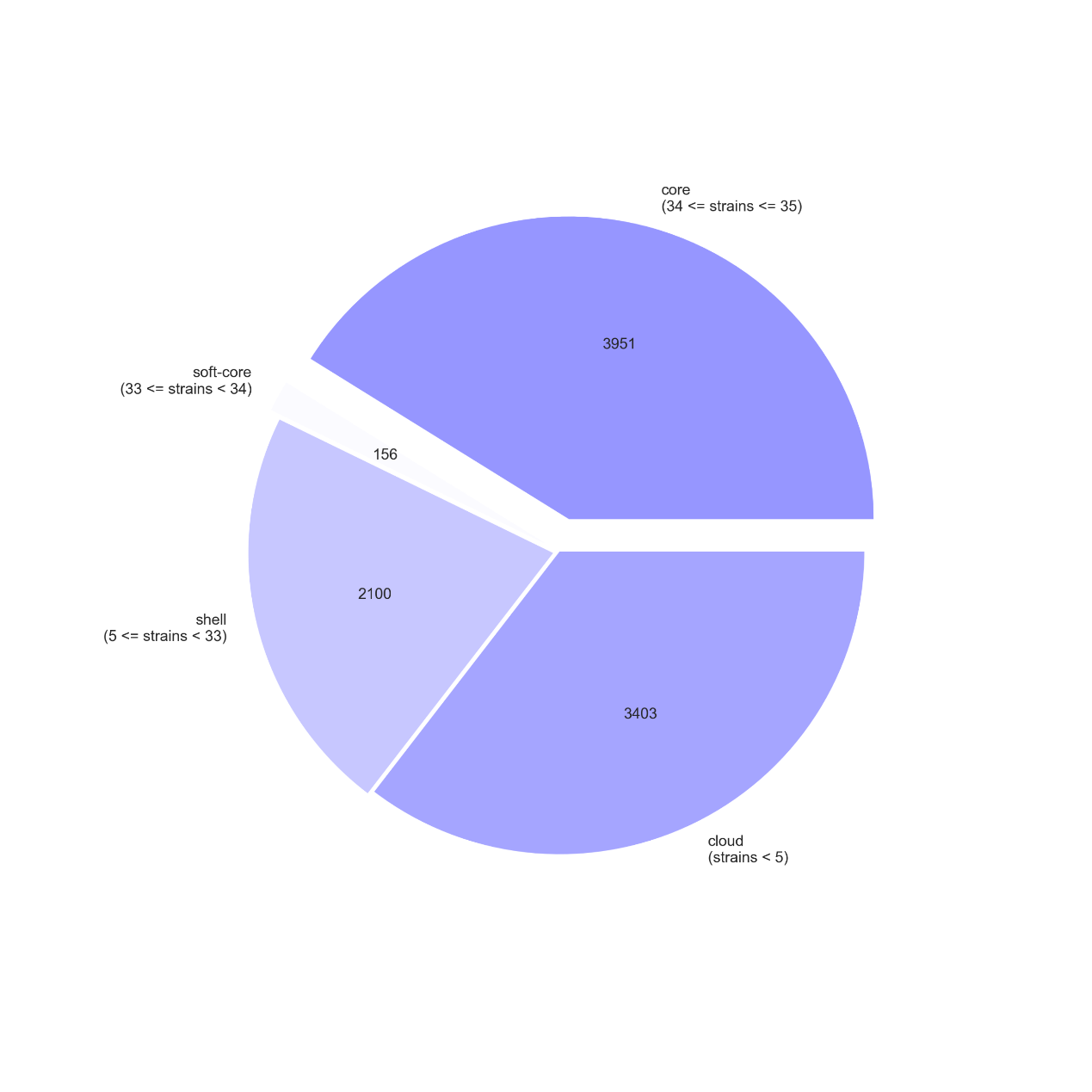


**Figure S2. The distribution of core genes, soft core genes, shell genes, and cloud genes.** The shade of color represents the abundance of corresponding genes.


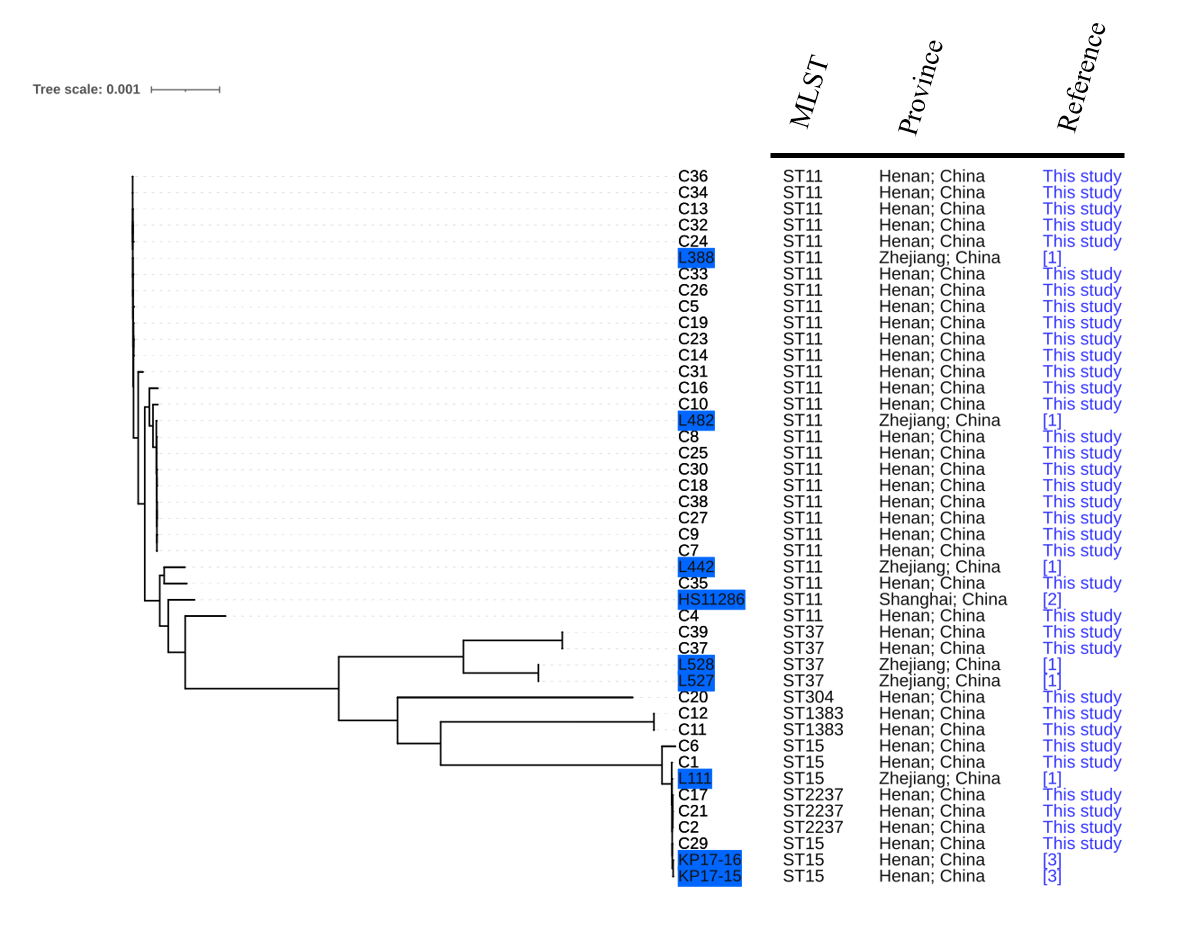


**Figure S3. Phylogenetic analysis between 35 isolates in this study and other *K. pneumoniae* strains.** The labels marked in blue represented the *K. pneumoniae* strains reported in other studies. Reference: [1] (Zheng et al., 2020), [2] (Liu et al., 2012), [3] (Li et al., 2020).


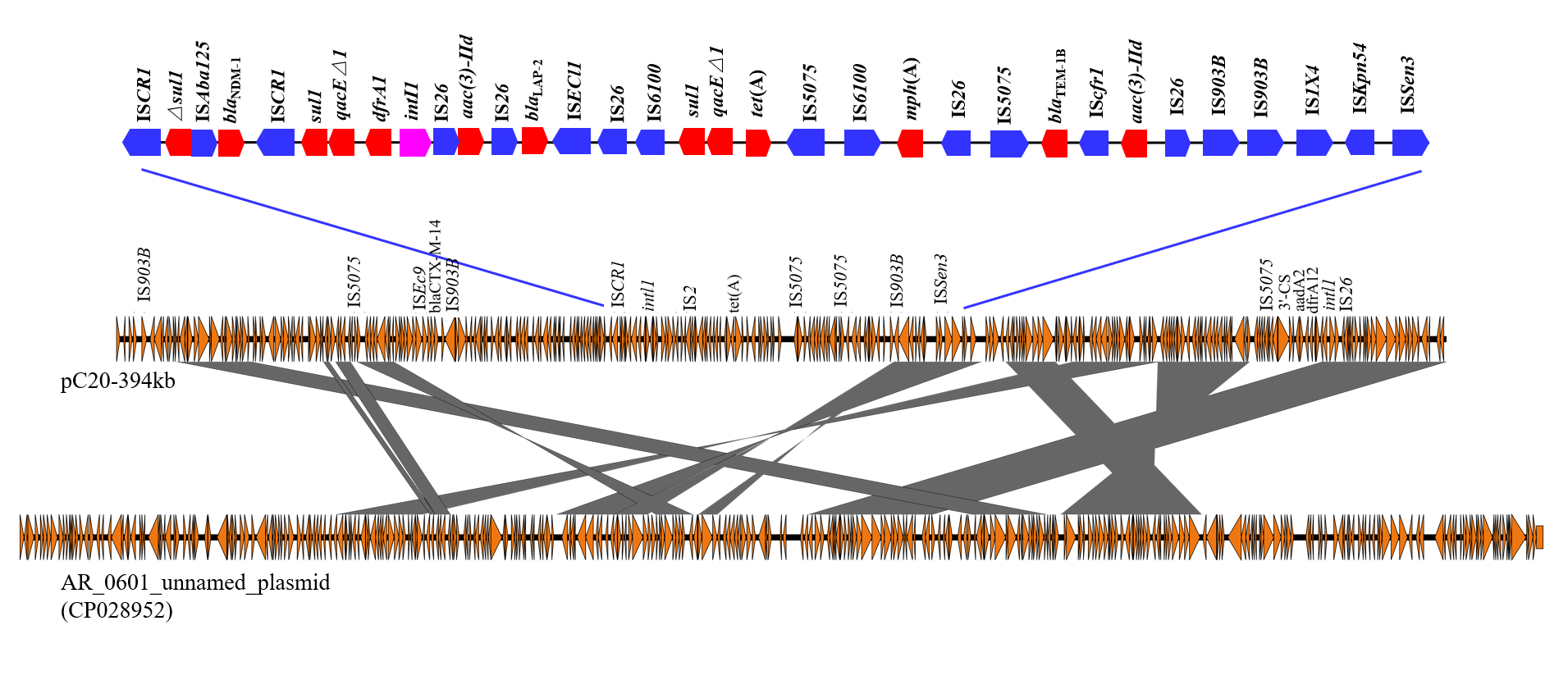


**Figure S4.** **Linear comparison of complete sequences between pC20-394kb in this study and AR_0601_unnamed_plasmid (CP028952).** The diagram of the arrangement of AMR genes and insertion sequences in MDR region of pC20-394kb is presented at the top of the figure.


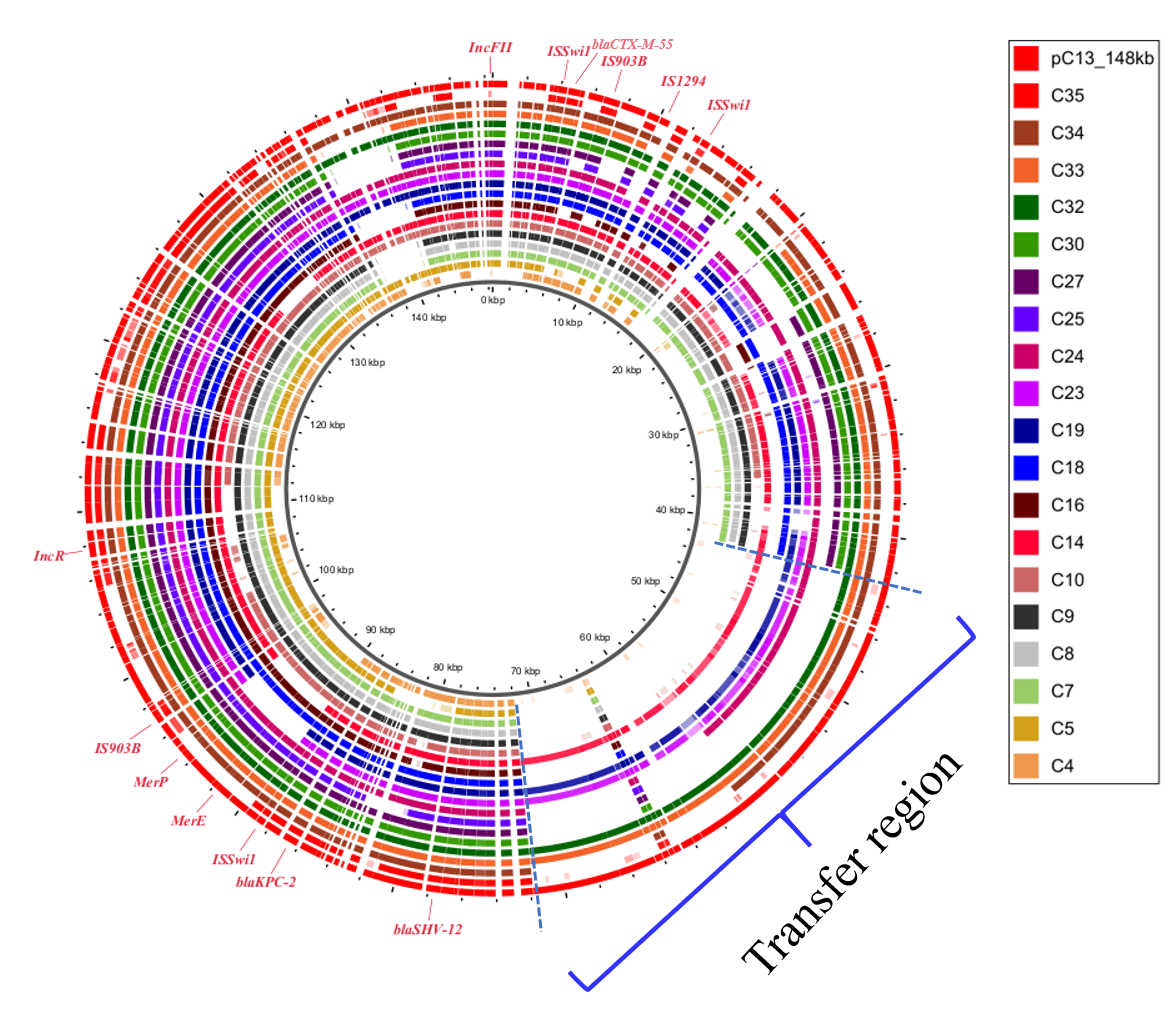


**Figure S5. Circular comparison between the pC13_148kb and the assembled contigs based on Illumina data of other 19 ST11 CRKP isolates in this study.**

**References**

Li, R., Cheng, J., Dong, H., Li, L., Liu, W., Zhang, C., Feng, X., and Qin, S. (2020). Emergence of a novel conjugative hybrid virulence multidrug-resistant plasmid in extensively drug-resistant Klebsiella pneumoniae ST15. *Int J Antimicrob Agents* 55**,** 105952.

Liu, P., Li, P., Jiang, X., Bi, D., Xie, Y., Tai, C., Deng, Z., Rajakumar, K., and Ou, H.Y. (2012). Complete genome sequence of Klebsiella pneumoniae subsp. pneumoniae HS11286, a multidrug-resistant strain isolated from human sputum. *J Bacteriol* 194**,** 1841-1842.

Poirel, L., Walsh, T.R., Cuvillier, V., and Nordmann, P. (2011). Multiplex PCR for detection of acquired carbapenemase genes. *Diagn Microbiol Infect Dis* 70**,** 119-123.

Yu, F., Lv, J., Niu, S., Du, H., Tang, Y.W., Pitout, J.D.D., Bonomo, R.A., Kreiswirth, B.N., and Chen, L. (2018). Multiplex PCR Analysis for Rapid Detection of Klebsiella pneumoniae Carbapenem-Resistant (Sequence Type 258 [ST258] and ST11) and Hypervirulent (ST23, ST65, ST86, and ST375) Strains. *J Clin Microbiol* 56.

Zheng, B., Xu, H., Lv, T., Guo, L., Xiao, Y., Huang, C., Zhang, S., Chen, Y., Han, H., Shen, P., Xiao, Y., and Li, L. (2020). Stool Samples of Acute Diarrhea Inpatients as a Reservoir of ST11 Hypervirulent KPC-2-Producing Klebsiella pneumoniae. *mSystems* 5.
